# Supplementary material for: Vaginal birth core information set: study protocol for a Delphi study to achieve a consensus on a ‘core information set’ for vaginal birth
Source: BMJ Open. 2023 Aug 7;13(8):e070215. doi: 10.1136/bmjopen-2022-070215 (PMC10407363; doi:10.1136/bmjopen-2022-070215)
Supplement: Supplementary data [file bmjopen-2022-070215supp001.pdf]

Core Outcome Set-STAndardised Protocol Items (COS-STAP) Checklist for the 'Vaginal Birth Core Information Set' Study

Item Number	Name	Location
1	Identify in the title that the paper describes the protocol for the planned development of a CIS	Title/Abstract<br>Page 1-2
2	Provide a structured abstract	Abstract<br>Page 2-3
3	Describe the background and explain the rationale for developing the CIS	Background<br>Page 4
4	Describe the specific objectives with reference to developing a CIS	Background and Aim<br>Page 4-6
5	Describe the health condition(s) and population(s) that will be covered by the CIS	Methods/Design (Scope of CIS)<br>Page 7
6	Describe the intervention(s) that will be covered by the CIS	Methods/Design<br>Page 7
7	Describe the setting(s) that will be covered by the CIS	Methods/Design<br>Page 7
8	Indicate the CIS study registration details and registry name. If not yet registered indicate the intended registry	Methods/Design<br>Page 7
9	Describe any study oversight committees	Oversight and monitoring (Trial steering committee)<br>Page 18
10	Describe sources of funding, role of funders	Declarations (Funding)<br>Page 20
11	Describe any potential conflicts of interest within the study team and how these will be managed	Declarations (Competing interests)<br>Page 20
12	Describe the stakeholder groups to be involved in the CIS development process and the rationale for their involvement	Oversight and monitoring (Trial steering committee)<br>Page 18
13	Describe the eligibility criteria for individuals from each stakeholder group	Study overview<br>Page 10-14
14	Describe how individuals of each stakeholder groups will be identified	Study overview<br>Page 10-14

15	Describe how individuals of each stakeholder group will be chosen from within the stakeholder group	Study overview<br>Page 10-14
16	Describe how many planned individuals within each stakeholder group will be invited to participate in the consensus process	Study overview<br>Page 10-14
17	Describe how individuals will be invited to take part in the consensus process	Study overview (Stage 4)<br>Page 16
18	Describe the information sources that will be used to identify the list of outcomes. Outline the methods or reference other protocols/papers.	Study overview (Stage 1)<br>Page 8-11
19	Describe how outcomes may be dropped/combined, with reasons	Study overview Study overview (Stage 2)<br>Page 12
20	Describe the methods to identify outcome descriptor terms	Study overview (Stage 2)<br>Page 12
21	Describe the plans for how the consensus process will be undertaken	Study overview (Stage 4)<br>Page 16
22	Describe what information will be presented to participants at the start of the consensus process	Study overview (Stage 4)<br>Page 16
23	Describe what each participant will be asked to do at each stage of the consensus process	Study overview (Stage 4)<br>Page 16
24	Describe how the participants will receive any feedback during the consensus process	Study overview (Stage 4)<br>Page 16-17
25	Describe how non-response (or partial response) will be handled during the consensus process	Study overview (Stage 4)<br>Page 16
26	Describe how the study material will be made patient friendly and understandable (if relevant)	Study overview (Think-aloud interviews)<br>Page 12
27	Describe the consensus definition	Study overview (Delphi rounds)<br>Page 15
28	Describe the procedure for determining how outcomes will be added/combined/dropped from consideration during the consensus process	Study overview (Delphi rounds)<br>Page 15
29	Describe how outcomes will be scored and summarised	Study overview (Delphi rounds)<br>Page 15

30	Describe how the response rate will be maximised	Study overview (Piloting the Delphi survey)<br>Page 13
31	Describe how attrition bias will be assessed	Study overview<br>Page 16-17
32	Describe any software that will be used during the consensus process and to analyse the results	Study overview<br>Page 14
33	Describe any plans for obtaining research ethics committee / institutional review board approval in relation to the consensus process (if relevant)	Declarations (Ethics approval and consent to participate)<br>Page 19
34	Describe how informed consent will be obtained (if relevant)	Study overview<br>Page 12-17
35	Describe any details about how the confidentiality of data collection will be preserved during the consensus process (if relevant)	Study overview<br>Page 16-17
