# Supplementary material for: Vaginal birth core information set: study protocol for a Delphi study to achieve a consensus on a ‘core information set’ for vaginal birth
Source: BMJ Open. 2023 Aug 7;13(8):e070215. doi: 10.1136/bmjopen-2022-070215 (PMC10407363; doi:10.1136/bmjopen-2022-070215)
Supplement: Author's manuscript [file bmjopen-2022-070215.draft_revisions.pdf]

BMJ Open

BMJ Open is committed to open peer review. As part of this commitment we make the peer review history of every article we publish publicly available.

When an article is published we post the peer reviewers' comments and the authors' responses online. We also post the versions of the paper that were used during peer review. These are the versions that the peer review comments apply to.

The versions of the paper that follow are the versions that were submitted during the peer review process. They are not the versions of record or the final published versions. They should not be cited or distributed as the published version of this manuscript.

BMJ Open is an open access journal and the full, final, typeset and author-corrected version of record of the manuscript is available on our site with no access controls, subscription charges or pay-per-view fees (http://bmjopen.bmj.com).

If you have any questions on BMJ Open's open peer review process please email info.bmjopen@bmj.com

BMJ Open

Vaginal Birth Core Information Set: Study protocol for a Delphi study to achieve a consensus on a 'core information set' for vaginal birth

Journal:	*BMJ Open*
Manuscript ID	bmjopen-2022-070215
Article Type:	Protocol
Date Submitted by the Author:	17-Nov-2022

Complete List of Authors:	Demetri, Andrew; University of Bristol,<br>Davies, Anna; University of Bristol<br>Bakhbakhi, Danya; University of Bristol<br>Ijaz , Sharea ; University of Bristol<br>Dawson, Sarah; University of Bristol<br>McGuinness, Sheelagh; University of Bristol<br>Beasor, Gemma; University of Bristol<br>Clayton, Gemma; University of Bristol<br>Johnson, Abigail; North Bristol NHS Trust<br>De Souza, Chloe; North Bristol NHS Trust<br>Dempsey, Aine; University Hospitals Bristol and Weston NHS Foundation Trust<br>Snook, Gabriella; North Bristol NHS Trust<br>Sharp, Andrew; University of Liverpool<br>Lissauer, David ; University of Liverpool<br>Mcgoldrick, Emma; Liverpool Women's Hospital<br>Burden, Christy; University of Bristol<br>Merriel, Abi; University of Liverpool

Keywords:	OBSTETRICS, Maternal medicine < OBSTETRICS, Anaesthesia in obstetrics < ANAESTHETICS, MEDICAL ETHICS

SCHOLARONE™
Manuscripts

Title: Vaginal Birth Core Information Set: Study protocol for a Delphi study to achieve a consensus on a 'core information set' for vaginal birth

Authors

Dr Andrew Demetri, Academic Clinic Fellow, University of Bristol, andrew.demetri@bristol.ac.uk, **https://orcid.org/0000-0002-2820-5919**

Dr Anna Davies, Research Fellow, University of Bristol, anna.davies@bristol.ac.uk, **https://orcid.org/0000-0003-0743-6547**

Dr Danya Bakhbakhi, NIHR Doctoral Research Fellow, University of Bristol, danya.bakhbakhi@bristol.ac.uk

Dr Sharea Ijaz, Senior Research Associate in Evidence, University of Bristol, s.ijaz@bristol.ac.uk

Ms Sarah Dawson, Senior Research Associate in Information Retrieval, University of Bristol, sarah.dawson@bristol.ac.uk

Professor Sheelagh McGuinness, Reader in Law, University of Bristol, sheelagh.mcguiness@bristol.ac.uk

Ms Gemma Beasor, Patient representative, gemma.beasor@hotmail.co.uk

Dr Gemma Clayton, Senior Research Associate in Biostatistics, University of Bristol, gemma.clayton@bristol.ac.uk

Ms Abigail Johnson, Midwife, North Bristol NHS Trust, Abigail.Johnson@nbt.nhs.uk

Dr Chloe de Souza, Obstetrics and Gynaecology Trainee, North Bristol NHS Trust, chloe.desouza@nbt.nhs.uk

Dr Aine Dempsey, Obstetrics and Gynaecology Trainee, St Michael's Hospital, aine.dempsey@nhs.net

Dr Gabriella Snook, Obstetrics and Gynaecology Trainee, North Bristol NHS Trust, gabriella.snook@nbt.nhs.uk

Dr Andrew Sharp, Senior Clinical Lecturer in Obstetrics, University of Liverpool, asharp@liverpool.ac.uk

Professor David Lissauer, NIHR Professor of Global and Fetal Medicine, University of Liverpool, David.Lissauer@liverpool.ac.uk

Dr Emma McGoldrick, NIHR Research Scholar and Consultant Obstetrician, Liverpool Women's Hospital, EmmaLouise.McGoldrick@lwh.nhs.uk

For peer review only

For peer review only - http://bmjopen.bmj.com/site/about/guidelines.xhtml

Dr Christy Burden, Consultant Senior Lecturer, University of Bristol, christy.burden@bristol.ac.uk

Dr Abi Merriel, Senior Clinical Lecturer in Obstetrics, University of Liverpool, abi.merriel@liverpool.ac.uk,

https://orcid.org/**0000-0003-0352-2106**

Corresponding author

Name: Dr Abi Merriel

Role: Senior Clinical Lecturer in Obstetrics

Postal address: University of Liverpool

Tel: 07740334922

Email: abi.merriel@liverpool.ac.uk

Word count: 3,968

For peer review only

Abstract

Introduction

Studies have shown that women are often underinformed about potential benefits and risks of vaginal birth. This is in contrast to other modes of delivery, such as caesarean delivery, for which the risks/benefits are often conveyed prior to undergoing the procedure. A core information set (CIS) is an agreed set of information points that should be discussed with a patient prior to undergoing a procedure or intervention. This will improve the quality of information given regarding mode of birth. This CIS will mean women can be given the information prioritised by patients and stakeholders regarding vaginal birth, empowering them to make informed decisions about their birth. We aim to describe the protocol for the development of this CIS about vaginal birth.

Methods and analysis

We will develop the CIS by:

1. Compiling a long list of information points about vaginal birth by: undertaking a scoping review of studies and patient information leaflets; interviews with antenatal and postnatal women, and an online survey of stakeholders.

2. Collating a 'long list' of information points from the scoping review and interviews and developing the Delphi survey. Think-aloud interviews will refine this prior to launch.

3. Conducting an online two-round Delphi survey. 200 stakeholder participants will be recruited. Items rated as critically important by ≥80% of participants in one stakeholder group, or for which there is no consensus, will be carried through to a stakeholder consensus meeting to decide the final CIS.

For peer review only

Discussion

We will use a systematic approach to identify information items, and to achieve consensus, with the input of a wide range of stakeholders, ensuring the CIS is useful and relevant to clinical care and women's informational needs. The CIS will be used to inform clinical conversations and patient information leaflets/websites, enabling women to make informed decisions about vaginal birth.

Registration

This study is registered through the COMET core outcome set initiative:

https://www.comet-initiative.org/Studies/Details/2069

Ethics and dissemination

This project has been given a favourable ethics opinion by the University of Bristol Research Ethics Committee (Ref: 10530).

If amendments to the protocol and/or the study documents are required, relevant approvals will be sought from the ethics committee. The principal investigator will be responsible for decisions to amend the protocol and to determine whether the amendment is substantial or non-substantial. Relevant study documents will be altered, and the changes made tracked to demonstrate where they have been made.

Amendments to the protocol will be documented using sequential version numbers, with updated documents replaced in the site file.

For peer review only

We will present the CIS at relevant conferences and will produce a publication of the final CIS for a relevant high-impact journal. We will disseminate the CIS via our partner, Policy Bristol, with the aim to share it with clinical policy and guideline developers.

Key words

Core information set, Delphi technique, Consensus, Informed consent, Vaginal birth, Stakeholders, Women, Women's health

Article Summary

Strengths and limitations of this study

- This will be the first published core information set regarding vaginal birth, which will be able to be utilised in future work to improve discussions women have with their healthcare practitioners, ultimately leading to women and their partners receiving better quality information regarding their birth choices, improving their birth experiences.
- Patient involvement is central to the development of this body of work, meaning that the core information set will reflect the beliefs and thoughts of the women it will be helping. Women's voices will be key at each stage of developing the core information set.
- These methods, including the use of in-depth qualitative interviews, the Delphi survey and consensus process, means the methods for the development of this core information set will be robust and comprehensive, adding weight to the findings.
- The qualitative interviews, Delphi survey and consensus meetings will only be conducted in the English language due to funding and time limitations. Future research may be used to explore differences in core information regarding vaginal birth in different languages and cultures.

For peer review only

- Due to time and financial constraints, in-depth qualitative interviews will only include women who have given birth in the last 5 years, so potentially may miss some longer-term information points relating to vaginal birth. However, use of the scoping review of literature and stakeholder survey to identify information points should mitigate this limitation, by identifying longer term outcomes.

For peer review only

Background

Spontaneous vaginal birth accounts for approximately 50% of births in the United Kingdom.[1] Its benefits, risks and range of experiences are often not discussed in detail with women prior to them opting for a vaginal birth. This is perhaps because vaginal birth is the presumed choice for women, unless they have had a pregnancy- or previous birth-related indication for, or request, a caesarean section (CS). To improve the quality of maternity care, women must be provided with the information they require to make a fully informed choice about their planned mode of birth.

Vaginal birth (VB) is a physiological process and has benefits for mother and baby. Examples include shorter hospital stays, quicker recovery times and increased rates of breast feeding compared with CS.[2,3] Risks of VB include shoulder dystocia (when a baby's shoulders get stuck once its head has been born) which may lead to birth trauma or asphyxia, and 3rd or 4th degree tears which can result in longer term pelvic floor dysfunction and pain.[4] These outcomes may have significant impact on women's longer term physical and psychological functioning.[5]

However, even if a woman has made a positive informed choice for a VB, it may not be successful. In 2020-1, NHS statistics showed that 15% of women required an emergency instrumental delivery, and 19% an emergency CS, and most, although not all, of these will have been aiming for a VB.[6] The risks of emergency CS are increased compared with elective CS, such as having a higher incidence of wound infection and postpartum haemorrhage (PPH).[4,7] Likewise, an instrumental delivery carries higher risks than spontaneous vaginal birth, including increased risk of perineal trauma.[4,7] Knowledge about VB, including the risks

For peer review only

and benefits, may influence women's thoughts and choices on their mode of delivery and birth experience. However, women are generally not explicitly offered information or this choice.

There is a lack of, and inconsistency in, the information that women receive about VB.[2] This lack of information antenatally can contribute to poorer birth experiences by increasing anxieties with 'fear of the unknown'.[8,9] Good clinical practice and NHS improvement support the provision of information to patients to enable them to make truly informed decisions.[11,12] Furthermore, the landmark Montgomery case highlighted the importance of patient focused risk disclosure.[10] By not fulfilling these criteria, healthcare professionals are failing in the professional, moral, and legal requirement, to support decision making about birth.

Women need access to consistent, accurate information, about what is important to them, when making birth choices. Whilst there has been work looking at improving information provision and outcomes that are important to women surrounding CS,[13] very little has been done to understand what information women need about vaginal birth to decisions about mode of delivery. Core information sets (CIS) have been developed as a systematic means to provide information that is judged to be important by all relevant stakeholders about medical procedures. A CIS [14,15] is a list of key information points regarding an experience that should be discussed with a person prior to experiencing an event or procedure. They are developed with the input of key stakeholders including the patient, clinicians, and other relevant stakeholders, to identify the most important information to include.[16] Having a list to discuss with pregnant women will ensure that they are well informed about the decision they are

For peer review only - http://bmjopen.bmj.com/site/about/guidelines.xhtml

making. Previous CIS have been used as catalysts for discussion about procedures, to improve content of information leaflets and improve decision aids.[14]

We aim to produce a CIS for VB that can be used to facilitate informed choice by improving discussions and enhancing tools used to help women with decision-making about birth choices. This will support their preparation for vaginal birth, enable better birth experiences and greater satisfaction, as well as enhancing women's post-natal wellbeing. Here we describe the protocol for development of the CIS.

Aim

To develop a CIS for vaginal birth to support informed decision making for childbirth.

Methods/Design

Design

We will conduct a systematic scoping review, qualitative interviews, and use a modified Delphi technique to identify the key information for the CIS. The Delphi process has been used in prior CIS studies,[14,15] and involves multiple survey rounds of key stakeholder groups to condense the long list of information points, reaching a consensus on a CIS for vaginal birth.

Methods

This project has been given a favourable ethics opinion by the University of Bristol Research Ethics Committee (Ref: 10530).

For peer review only

Registration and reporting: The study is registered on the COMET website https://comet-initiative.org/Studies/Details/2069 and reporting adheres to the Core Outcome Set-STAndards for Development (COS-STAD) and COS-STAndardised Protocol Items (COS-STAP) have been used to guide the development of the methods for this study.[17,18] A modified COS-STAP checklist for the vaginal birth CIS is included in the supplementary information (appendix S1). The COS-Standards for Reporting (COS-STAR) will used to report CIS.[19]

There is no standardised way to develop a CIS,[20] existing CIS have adapted methods from the development of core outcome sets.[14,15] The Core Outcome Measures in Effectiveness (COMET) handbook and methods from existing CIS have been used to design this study.[14–16,21]

SCOPE OF THE CIS

The CIS will be applicable to women who are considering spontaneous vaginal birth (SVD). SVD is an unassisted vaginal birth, without vacuum or forceps, with a natural onset not through medical intervention.[22,23] We therefore plan to exclude information points pertaining to the decision-making process of induction of labour, instrumental delivery, caesarean birth and vaginal birth after caesarean section (VBAC).

Patient and Public Involvement (PPI)

PPI is key to this study, with involvement of wide range of stakeholder groups in the development and undertaking of this study. Women's perspectives, and those of their partners, are pivotal to each stage of this study, including the qualitative interviews, Delphi survey and development, and consensus meeting. The study steering committee also

For peer review only

contains patient representation, ensuring that the development of the project is guided by those that the work will affect.

Study Overview

The development of this core information set will take place in four stages (figure 1: study overview diagram).

Stage 1: Identification of key information points.

A long list of information points pertaining to vaginal birth will be generated through three methods:

a) Scoping review of the literature: Information points about vaginal birth from published studies will be extracted as well as from patient information leaflets provided to women in the NHS and other trusted organisations.
b) Interviews with women: qualitative interviews will be conducted with pregnant and postnatal women to determine what information is valued by women
c) Stakeholder survey: An online survey of stakeholders (women, partners, healthcare professionals, interested groups and medico-legal experts) to identify what information is thought to be of importance to women

a) Scoping review

Search: Information about vaginal birth will be identified from systematic reviews of pregnancy and childbirth literature and supplemented by a review of patient information leaflets. A pragmatic search strategy will be developed by an information specialist to identify outcomes reported in studies, and key issues women identify, about vaginal birth.

For peer review only

Studies included in the review will be systematic reviews, both qualitative and quantitative. Search terms will include vaginal birth, vaginal delivery, birth, childbirth, natural childbirth, labour, labor, parturition. Searches will be applied to the following electronic databases: CINAHL, Embase, MEDLINE, Web of Science, DoPHER, Epistemonikos, Health Evidence and the Cochrane Library. Searches will be limited to systematic reviews published in the English language. The date is limited to 2020 to 2022 for the papers, and to 2017 to 2022 for the Cochrane reviews, due to the large volume of papers that would meet the criteria, thus meaning data saturation from the literature is likely to be achieved within this sample.

We will also search for patient information leaflets on vaginal birth, to extract information points from these. We will search the online leaflet collections of the Royal College of Obstetricians and Gynaecologists, Tommy's Pregnancy Information and NHS trusts with an online database of leaflets.

Paper selection: Screening of titles, abstracts and full texts will be carried out by two members of the research team using systematic reviewing software (Covidence).[24] Any disagreements will be resolved by a third reviewer. We will include all qualitative, quantitative, and mixed-method systematic reviews that include information points or outcomes for vaginal birth. No studies will be excluded based on judgement methodological quality or risk of bias assessment as the purpose of the scoping review is to identify information points and outcomes relating to vaginal birth, not to examine the quality of data.

Data extraction: Data will be extracted using a pre-designed form, piloted by two members of the research team on a sample of included studies. Data will include basic publication

For peer review only

For peer review only - http://bmjopen.bmj.com/site/about/guidelines.xhtml

289 details such as author, date of publication, study setting and methodology. A pre-designed, and piloted, form will also be used for data extraction from leaflets. Extracted data will include format of leaflet, source, and year of publication. Outcomes and information points will be extracted verbatim. It will not be necessary to conduct a risk of bias analysis.

b) Qualitative interviews

Method: Semi-structured interviews

Sample: We will recruit up to 20 women who are >12 weeks pregnant, and/or women who have had a baby, including those who have experienced an intrauterine death, in the last 24 months. Sample size for these interviews will be determined by data saturation being reached, whereby interviews in each group of participants will end when no new themes are generated. Women will be recruited online, via social media and by adverts in community centres where these are possible to display. We will purposively select women with a range of birth experiences or birth preferences including vaginal birth, caesarean birth, and assisted vaginal birth. This will ensure that we gather information points from women from diverse stand points in terms of their decision-making journey vaginal birth. Those who are unable to speak English or consent to interview will be excluded from recruitment.

Interviews: Informed consent will be gained using an online form, hosted in REDCap.[25] A topic guide will be developed with the study steering group (appendix S2). Interviews will be conducted by a qualitative researcher, recorded on an encrypted audio recorder uploaded on to the secure University of Bristol server. At the start of the interview, demographic questions will be asked. Participants will receive a £10 voucher for to compensate for their time and inconvenience.

For peer review only

Analysis: Audio recording of the interviews will be transcribed, and analysed using NVivo software, using thematic analysis.[26] Two members of the research team will read the transcripts initially to familiarise themselves with the process. Interviews will be independently coded by the two members of the research team, to identify important information points about vaginal birth from the words of participants. Transcripts will be re-read and each researcher's codes compared for each interview. Codes will then be grouped to create key themes, and key information points will be identified from each of these themes.[27] Analysis whilst the interviews are underway will ensure that when saturation of themes is reached, no additional interviews will be conducted unnecessarily.[28–30]

c) Stakeholder survey:

Method: An online qualitative survey will be used to identify further information items by gathering key stakeholder views on what information is important for women to receive about vaginal birth. Sample: Stakeholders include antenatal and postnatal women, their partners, healthcare professionals who work alongside women in labour and postnatally, representatives from groups who have an interest in women's birthing rights and medico-legal experts with an interest in childbirth. We will aim to engage approximately 200 survey participants, recruited online through social media and in community spaces. This sample size has been decided upon to ensure that a wide range of views and experiences are captured. We will aim for half of the responses to be from parents whose experiences and views are central to this work.

For peer review only

For peer review only - http://bmjopen.bmj.com/site/about/guidelines.xhtml

Data collection: The survey will be carried out online using REDCap.[25] The survey will include information on the participant's role and demographics, including if they have previously experienced a vaginal birth.

To identify what information they believe is vital to provide to women about vaginal birth, a free text box will be used. All participants in the survey will also be invited to participate in the Delphi process. Participants in the survey will be entered into a prize draw to win a voucher worth £10 (5 vouchers available).

Analysis: Survey data will be thematically analysed by two researchers and key information points extracted. These information points will be added to those from the scoping review and interviews.

Stage 2: Developing the long list of information points and Think-aloud Interviews

Developing the long list of information points

Following the identification of key points, we will produce an exhaustive list of information items relating to vaginal birth, known as a 'long list', which will feed into the initial stage of the Delphi process.[31,32] Two members of the team will use all information items from the scoping review, interviews, and stakeholder survey to generate the comprehensive list. Information items will be organised into categories for coherence. Examples of categories for information points which may be included are for points relating to maternal physical health, maternal mental health, baby health, admission, procedures. Categories may be removed or created dependent on the information points that are discovered.

A meeting will be held to finalise the long list for the Delphi process and establish how the items should be presented and worded in the Delphi survey. This meeting will involve members of the research team. During this meeting, categories will be discussed for each item. Additionally, definitions will be decided upon to ensure they are understandable for all survey participants. Where items are similar, group discussions and decisions will take place to ensure there is no duplication.

Piloting the Delphi survey

Up to 10 think-aloud interviews will be undertaken to test the readability and usability of the Delphi survey and refine the long list of information items.[33] The think-aloud method is a well-recognised validation technique for developing an instrument or tool, and has been used by COS developers to improve their Delphi questionnaires.[16,34] This method is a form of cognitive interviewing that asks participants to verbalise their thoughts as they engage with a task, in this case the Delphi survey.[35] We will observe how participants interpret the information items and definitions, and how they interact with the scoring system, identifying any issues. Participants thoughts are then used to improve and perfect the survey to ensure its usability to ensure it is understandable and easy to complete. We will also record the length of time the survey takes, to ensure that it is not too long, therefore minimising answer fatigue and maximising responses.[16]

After completing a consent form, participants will be asked to trial the Delphi survey using the online platform. Interviews will last thirty minutes to one hour and may take place in person or online via teleconferencing software. The interviews will be audio recorded on a University of Bristol (UoB) encrypted device, and the interviewer will take notes. 2-3 initial interviews will take place, and comments regarding the survey and possible improvements

For peer review only - http://bmjopen.bmj.com/site/about/guidelines.xhtml

will be noted, with the audio recordings only used if needed to remind about key points. This noted improvements will then be used to improve the survey, before carrying out the next 2-3 surveys and repeating the process. The approach is repeated until no further changes are indicated, which we estimate will be after approximately 10 interviews.

Stage 3: The Delphi Survey

Delphi overview

We will aim to conduct two iterative Delphi rounds, followed by a consensus meeting. The survey will be conducted online using REDCap hosted by the University of Bristol.[25]

Participants and sample size

Participants in the Delphi process will include:

1) Women and their partners:

   Any woman, who is over the age of 18, who is currently pregnant, or has had a baby in the last 5 years, and partners of these women.

2) Healthcare professionals

   Those who work with, or provide care for women, during their pregnancy, during childbirth, and/or postnatally (e.g. obstetricians and gynaecologists include those in training, midwives, midwifery support workers, physiotherapists, anaesthetists).

3) Medicolegal experts

   Legal professionals who have a specialist interest in reproductive health.

4) Representatives from interested groups/stakeholders

   Representatives from organisations and groups who have an interest in women's birthing rights (e.g. Birthrights, Maternity Action).

For peer review only

Sample size: We will obtain a sample with a broad range of experience, an approach supported by the COMET guidelines and previous studies.[14,21] There is no specified size of stakeholder sample for CIS or COS studies.[15,20,21] Some have estimated that the is a drop-out rate for a Delphi process is around 20%.[32] To account for this potential attrition, we will aim to engage at least 200 participants in the Delphi process. We will aim for at least half of these participants to be antenatal and postnatal women, as their views are vital for this CIS to be relevant.

For peer review only

Delphi rounds

A two-round modified Delphi process will be conducted online, with each round lasting for 2-8 weeks depending on level of participation received.[14,25] Participants will be asked to complete a consent process before completing the survey as well as demographic/ occupational information. Participants will score each survey item for importance for inclusion from 1-9 (1-3- limited importance; 4-6- important but not critical; 7-9- critical).[21,36] A-priori consensus criteria will be applied.[31] Information items to be carried through to the subsequent round will require ≥80% of participants from either patient/representative group (women, partners and representatives of interested groups) or the professional group (healthcare workers and medico-legal experts) to score it as critically important (7-9) and <15% from any group to classify it as limited importance. Items will not be carried forward if over 80% of one group believe it is of limited importance, and if less than 15% believe it is critically important. Where items are rated as critical by ≥80% of participants in one stakeholder group, or there is no consensus, they will be carried forward to the next round.

In round two participants who took part in the first survey will be asked to recomplete the survey. They will be informed of their own scores and the median scores of patient/representative and professional groups using both numeric data and graphical illustrations of theirs and others' responses. Items carried through to the consensus meetings will be selected based on the above criteria. If many of the information points meet the criteria to be carried forward to either the 2nd Delphi round or the consensus meeting, then steering group will meet to review the criteria.

In this situation, an adapted form of the OMECRACT onion/framework may be used to organise information items.[37,38] Based on the methodology used by OMERACT, if many information points have met the criteria to be included, items will be grouped into categories to determine their importance and need to be included in the final CIS. Concentric circles indicate the hierarchy of the information points discussed at the consensus meeting (figure 2: OMECRACT onion/framework may be used to organise information items). The first central ring will be 'absolutely vital information to be included in the CIS'. A further two groups for information points will be used, with the middle ring being 'information that is useful to have available but may not be vital for inclusion in the CIS' and an outer ring for items that 'may or may not need to be discussed dependent on the personal circumstances or preferences.'

We will determine the level of attrition bias by comparing the score of stakeholders who completed both Delphi rounds with the scores of those who completed only the first round.[16]

Stage 4: The consensus meeting to decide the final core information set

Consensus meeting

For peer review only - http://bmjopen.bmj.com/site/about/guidelines.xhtml

Following the second Delphi round, an online consensus meeting, using teleconferencing software, will take place with all participants from the second round of the Delphi survey invited. If a large number of the invited stakeholders accept the invitation, we will aim to have the minimum representation from each stakeholder group.[16] The aim will be to recruit 8-10 participants from each stakeholder group, as this number has been sufficient in the development of previous COS.[16,39] A modified nominal technique will be used as a template to drive these discussions and reach a group consensus.[40] This technique allows for *all* participants' views to be heard in the discussion.

During this meeting, items carried through from the second Delphi round will be discussed and descriptive statistics will be created for each domain and displayed to participants, to stimulate discussion, with median scores for each item presented as graphs.[1531]

The participants will anonymously rate the retained information domains from the second round using online voting software.[18] Items will be categorised as 'Consensus in- information point to be included in final CIS', 'Consensus out- item not included in the final CIS' or 'No consensus- items for which opinions on inclusion are divided'. Consensus will be defined as 80% or more of all participants agreeing on the vote for an item. Where consensus is not reached, there will be further discussion and additional voting. Scores of those participating in the consensus meeting will be compared to those who only participated in the first two Delphi rounds, to assess if the consensus meeting views are representative of those who only completed the survey.[16] When consensus is reached for all items, we will have the final CIS.

There is no definitive agreed amount of information points that should be included in a CIS. Of the CISs that have been published, the amount of information points that have been

For peer review only

included in the final CIS have ranged from 8 to 13.[14,15] We will therefore aim to have a maximum of 15 information points in our final CIS.

Discussion

Vaginal birth, is physiological and carries benefits but is not without risk. Informed consent requires that women are well informed when making birth choices. We will develop a CIS using a systematic, transparent, process to help to standardise the information given to women planning to have a vaginal birth. It will be informed by what women and other stakeholders believe is the most important information to be discussed, which, if taken up, will ensure all have equitable access to this information.

This CIS can be used as the catalyst for discussions about vaginal birth, to promote truly informed birth choices. With this CIS we will have worked out what information is important. Future work will be needed to elaborate on the information points, adding statistical data to support understanding of risks and benefits, from national databases, systematic literature reviews and expert input. The CIS can also be used to inform patient information leaflets and decision-making tools.

Trial status

This is protocol version 1.0, written on 19th August 2022.

Competing interests

The authors of this paper have no competing interest.

For peer review only

Funding

This study was funded by the Research England Policy Support Fund (PSF). The sponsors have played no role in designing the study, data collection, analysis, interpretation of data, writing of the report and decisions for publication.

Author contributions

ADe and AM conceived the study and obtained the funding. AD drafted the protocol and drafted the manuscript. AM, Ada, DB, CB, AS, DL and EM helped with drafting the manuscript and formulating the methods for the study. SJ, SI, SM, CdS, GC, ADemp, and GS assisted with conceiving stage 1. GB, GC, and AJ helped with study conception and feasibility. All authors read and approved the final manuscript.

Acknowledgements

We would like to thank the Research England Policy Support Fund (PSF) for funding this project.

Data statement

As this is a qualitative study, we do not intend on making the dataset publicly accessible. The data set will be put into the data.bris repository, with requests for access coming through this. The level of access will be controlled.

All members of the core study team will have access to this data. Individuals in the wider steering group will receive parts of the data, as required, for analysis and discussion, and can access the full dataset by visiting UoB.

For peer review only

It is not envisaged that this data will be used for analysis outside the remit of this study, however, we will ask permission to use the data for subsequent analysis in the consent form.

<u>List of abbreviations</u>

**CIS:**	Core information set
**VB:**	Vaginal birth
**CS:**	Caesarean section
**PPH:**	Postpartum haemorrhage
**COS-STAD:**	Core Outcome Set-STAndards for Development (COS-STAD)
**COS-STAP:**	COS-STAndardised Protocol Items (COS-STAP)
**COS-STAR:**	COS-Standards for Reporting (COS-STAR)
**VBAC:**	Vaginal birth after caesarean section

<u>Figures</u>

Figure 1: Study overview diagram. Diagram showing the individual stages of the study and how they feed into the next. Also demonstrates where PPI and stakeholder input will be key in the study.

Figure 2: Omeract onion framework. This diagram demonstrates the framework which framework may be used to organise information items discussed at the consensus meeting.

<u>Supplementary materials</u>

Appendix S1- COS-STAP Checklist

Appendix S2- Antenatal and postnatal interview topic guide

For peer review only

For peer review only - http://bmjopen.bmj.com/site/about/guidelines.xhtml

References

1. Maternity Services Monthly Statistics, February 2021 [Internet]. 2021 [cited 2022 Feb 21]. Available from: https://digital.nhs.uk/data-and-information/publications/statistical/maternity-services-monthly-statistics/february-2021
2. Shub A, Williamson K, Saunders L, McCarthy EA. Do primigravidae and their carers have a realistic expectation of uncomplicated labour and delivery? Australian and New Zealand Journal of Obstetrics and Gynaecology. 2012 Feb;52(1):73–7.
3. Sandall J, Tribe RM, Avery L, Mola G, Visser GH, Homer CS, et al. Short-term and long-term effects of caesarean section on the health of women and children. The Lancet [Internet]. 2018 Oct [cited 2022 Jul 11];392(10155):1349–57. Available from: https://www.nice.org.uk/about/what-we-do/our-programmes/nice-guidance/nice-guidelines/shared-decision-making
4. Gregory K, Jackson S, Korst L, Fridman M. Cesarean versus Vaginal Delivery: Whose Risks? Whose Benefits? Am J Perinatol. 2012 Jan 10;29(01):07–18.
5. Ford E, Ayers S. Post-Traumatic Stress Disorder following Childbirth. 2012.
6. NHS Digital. Maternity Services Monthly Statistics, February 2022, experimental statistics [Internet]. 2022 [cited 2022 Jun 21]. Available from: https://digital.nhs.uk/data-and-information/publications/statistical/maternity-services-monthly-statistics/february-2022-experimental-statistics
7. Darnal N, Dangal G. Maternal and Fetal Outcome in Emergency versus Elective Caesarean Section. J Nepal Health Res Counc. 2020 Sep 7;18(2):186–9.
8. Gammie N, Key S. Time's up! Women's experience of induction of labour. Pract Midwife. 2014 Apr;17(4):15–8.
9. Lavender T, Walkinshaw SA, Walton I. A prospective study of women's views of factors contributing to a positive birth experience. Midwifery. 1999 Mar;15(1):40–6.
10. Chan SW, Tulloch E, Cooper ES, Smith A, Wojcik W, Norman JE. Montgomery and informed consent: where are we now? BMJ. 2017 May 12;j2224.
11. Whitney SN. A New Model of Medical Decisions: Exploring the Limits of Shared Decision Making. Medical Decision Making. 2003 Jul 2;23(4):275–80.
12. Britain G. Maternity matters: choice, access and continuity of care in a safe service. Department of Health. 2007 Apr.
13. Briscoe KE, Haas DM. Developing a Core Outcome Set for Cesarean Delivery Maternal Infectious Morbidity Outcomes. Am J Perinatol. 2020 Mar 28;37(04):436–52.
14. Blazeby JM, Macefield R, Blencowe NS, Jacobs M, McNair AGK, Sprangers M, et al. Core information set for oesophageal cancer surgery. British Journal of Surgery. 2015 Jun 10;102(8):936–43.
15. McNair AGK, Whistance RN, Main B, Forsythe R, Macefield R, Rees J, et al. Development of a core information set for colorectal cancer surgery: a consensus study. BMJ Open. 2019 Nov 14;9(11):e028623.
16. Bakhbakhi D, Fraser A, Siasakos D, Hinton L, Davies A, Merriel A, et al. Protocol for the development of a core outcome set for stillbirth care research (iCHOOSE Study). BMJ Open. 2022 Feb 9;12(2):e056629.
17. Kirkham JJ, Davis K, Altman DG, Blazeby JM, Clarke M, Tunis S, et al. Core Outcome Set-STAndards for Development: The COS-STAD recommendations. PLoS Med. 2017 Nov 16;14(11):e1002447.

For peer review only

18. Kirkham JJ, Gorst S, Altman DG, Blazeby JM, Clarke M, Tunis S, et al. Core Outcome Set-STAndardised Protocol Items: the COS-STAP Statement. Trials. 2019 Dec 11;20(1):116.
19. Kirkham JJ, Gorst S, Altman DG, Blazeby JM, Clarke M, Devane D, et al. Core Outcome Set–STAndards for Reporting: The COS-STAR Statement. PLoS Med. 2016 Oct 18;13(10):e1002148.
20. Williamson PR, Altman DG, Blazeby JM, Clarke M, Devane D, Gargon E, et al. Developing core outcome sets for clinical trials: issues to consider. Trials. 2012 Dec 6;13(1):132.
21. Williamson PR, Altman DG, Bagley H, Barnes KL, Blazeby JM, Brookes ST, et al. The COMET Handbook: version 1.0. Trials. 2017 Jun 20;18(S3):280.
22. Prosser SJ, Barnett AG, Miller YD. Factors promoting or inhibiting normal birth. BMC Pregnancy Childbirth. 2018 Dec 18;18(1):241.
23. Clark RRS, Lake ET. Spontaneous vaginal birth varies significantly across US hospitals. Birth. 2021 Mar 10;48(1):44–51.
24. Covidence systematic review software. Melbourne, Australia: Veritas Healthth Innovation.
25. Harris PA, Taylor R, Thielke R, Payne J, Gonzalez N, Conde JG. Research electronic data capture (REDCap)—A metadata-driven methodology and workflow process for providing translational research informatics support. J Biomed Inform. 2009 Apr;42(2):377–81.
26. Braun V, Clarke V. Using thematic analysis in psychology. Qual Res Psychol. 2006 Jan;3(2):77–101.
27. Clarke V, Braun V. Teaching thematic analysis: Overcoming challenges and developing strategies for effective learning. Psychologist. 2013;26(2):120–3.
28. Patton MQ. Two Decades of Developments in Qualitative Inquiry. Qualitative Social Work. 2002 Sep 15;1(3):261–83.
29. Connelly LM, Peltzer JN. Underdeveloped Themes in Qualitative Research. Clinical Nurse Specialist. 2016 Jan;30(1):52–7.
30. Sandelowski M. Sample size in qualitative research. Res Nurs Health. 1995 Apr;18(2):179–83.
31. dos Santos F, Drymiotou S, Antequera Martin A, Mol B, Gale C, Devane D, et al. Development of a core outcome set for trials on induction of labour: an international multistakeholder Delphi study. BJOG. 2018 Dec;125(13):1673–80.
32. Hall DA, Smith H, Heffernan E, Fackrell K. Recruiting and retaining participants in e-Delphi surveys for core outcome set development: Evaluating the COMiT'ID study. PLoS One. 2018 Jul 30;13(7):e0201378.
33. Drennan J. Cognitive interviewing: verbal data in the design and pretesting of questionnaires. J Adv Nurs. 2003 Apr;42(1):57–63.
34. Messick S. Validity. 3rd ed. Linn R.L., editor. New York: American Council on Education/Macmillan Publishing; 1989. 13–104 p.
35. Ericsson KA, Simon HA. How to Study Thinking in Everyday Life: Contrasting Think-Aloud Protocols With Descriptions and Explanations of Thinking. Mind Cult Act. 1998 Jul;5(3):178–86.
36. Grade Working Group. GRADE [Internet]. [cited 2022 May 9]. Available from: http://www.gradeworkinggroup.org
37. Mackie SL, Twohig H, Neill LM, Harrison E, Shea B, Black RJ, et al. The OMERACT Core Domain Set for Outcome Measures for Clinical Trials in Polymyalgia Rheumatica. J Rheumatol. 2017 Oct;44(10):1515–21.

For peer review only

For peer review only - http://bmjopen.bmj.com/site/about/guidelines.xhtml

38. Humphrey-Murto S, Crew R, Shea B, Bartlett SJ, March L, Tugwell P, et al. Consensus Building in OMERACT: Recommendations for Use of the Delphi for Core Outcome Set Development. J Rheumatol. 2019 Aug;46(8):1041–6.

39. Perry H, Duffy JMN, Umadia O, Khalil A. Outcome reporting across randomized trials and observational studies evaluating treatments for twin-twin transfusion syndrome: systematic review. Ultrasound in Obstetrics & Gynecology. 2018 Nov;52(5):577–85.

40. Harvey N, Holmes CA. Nominal group technique: An effective method for obtaining group consensus. Int J Nurs Pract. 2012 Apr;18(2):188–94.

For peer review only

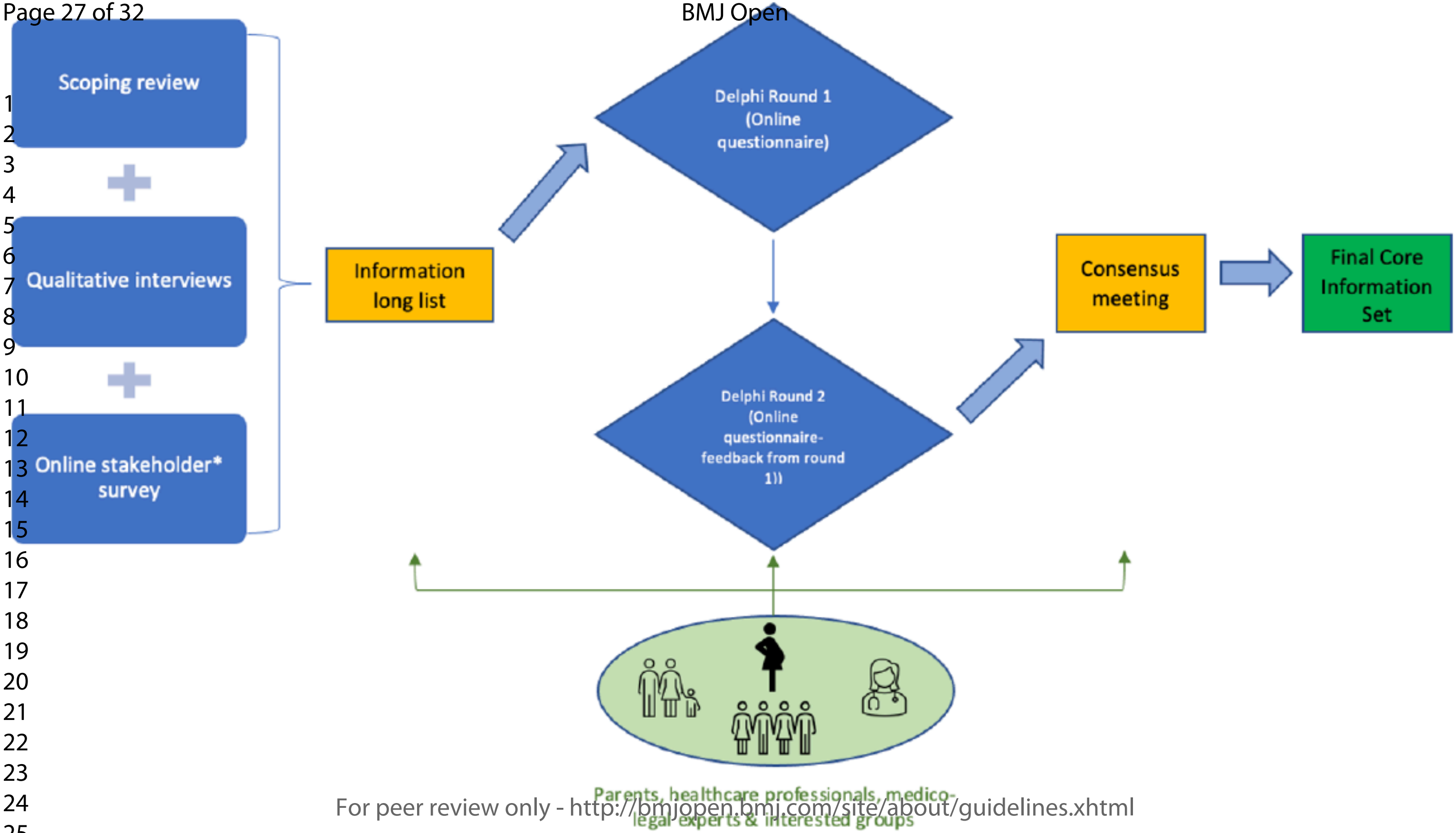

*Stakeholders include antenatal and postnatal women, their partners, healthcare professionals, interested groups and medico-legal experts

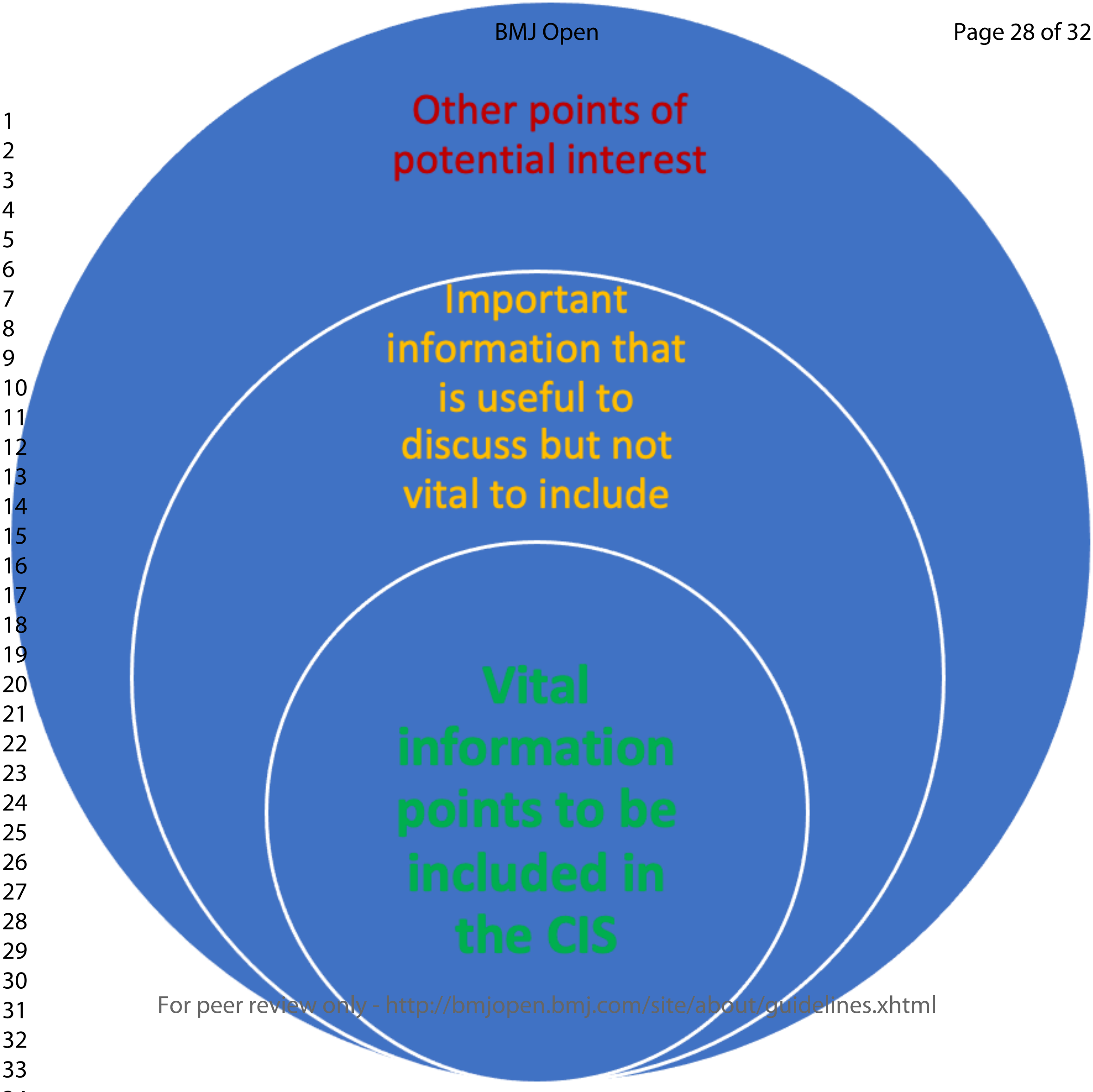

For peer review only - http://bmjopen.bmj.com/site/about/guidelines.xhtml

Core Outcome Set-STAndardised Protocol Items (COS-STAP) Checklist for the 'Vaginal Birth Core Information Set' Study

Item Number	Name	Location
1	Identify in the title that the paper describes the protocol for the planned development of a CIS	Title/Abstract<br>Page 1-2
2	Provide a structured abstract	Abstract<br>Page 2-3
3	Describe the background and explain the rationale for developing the CIS	Background<br>Page 4
4	Describe the specific objectives with reference to developing a CIS	Background and Aim<br>Page 4-6
5	Describe the health condition(s) and population(s) that will be covered by the CIS	Methods/Design (Scope of CIS)<br>Page 7
6	Describe the intervention(s) that will be covered by the CIS	Methods/Design<br>Page 7
7	Describe the setting(s) that will be covered by the CIS	Methods/Design<br>Page 7
8	Indicate the CIS study registration details and registry name. If not yet registered indicate the intended registry	Methods/Design<br>Page 7
9	Describe any study oversight committees	Oversight and monitoring (Trial steering committee)<br>Page 18
10	Describe sources of funding, role of funders	Declarations (Funding)<br>Page 20
11	Describe any potential conflicts of interest within the study team and how these will be managed	Declarations (Competing interests)<br>Page 20
12	Describe the stakeholder groups to be involved in the CIS development process and the rationale for their involvement	Oversight and monitoring (Trial steering committee)<br>Page 18
13	Describe the eligibility criteria for individuals from each stakeholder group	Study overview<br>Page 10-14
14	Describe how individuals of each stakeholder groups will be identified	Study overview<br>Page 10-14

For peer review only

15	Describe how individuals of each stakeholder group will be chosen from within the stakeholder group	Study overview<br>Page 10-14
16	Describe how many planned individuals within each stakeholder group will be invited to participate in the consensus process	Study overview<br>Page 10-14
17	Describe how individuals will be invited to take part in the consensus process	Study overview (Stage 4)<br>Page 16
18	Describe the information sources that will be used to identify the list of outcomes. Outline the methods or reference other protocols/papers.	Study overview (Stage 1)<br>Page 8-11
19	Describe how outcomes may be dropped/combined, with reasons	Study overview Study overview (Stage 2)<br>Page 12
20	Describe the methods to identify outcome descriptor terms	Study overview (Stage 2)<br>Page 12
21	Describe the plans for how the consensus process will be undertaken	Study overview (Stage 4)<br>Page 16
22	Describe what information will be presented to participants at the start of the consensus process	Study overview (Stage 4)<br>Page 16
23	Describe what each participant will be asked to do at each stage of the consensus process	Study overview (Stage 4)<br>Page 16
24	Describe how the participants will receive any feedback during the consensus process	Study overview (Stage 4)<br>Page 16-17
25	Describe how non-response (or partial response) will be handled during the consensus process	Study overview (Stage 4)<br>Page 16
26	Describe how the study material will be made patient friendly and understandable (if relevant)	Study overview (Think-aloud interviews)<br>Page 12
27	Describe the consensus definition	Study overview (Delphi rounds)<br>Page 15
28	Describe the procedure for determining how outcomes will be added/combined/dropped from consideration during the consensus process	Study overview (Delphi rounds)<br>Page 15
29	Describe how outcomes will be scored and summarised	Study overview (Delphi rounds)<br>Page 15

For peer review only

30	Describe how the response rate will be maximised	Study overview (Piloting the Delphi survey)<br>Page 13
31	Describe how attrition bias will be assessed	Study overview<br>Page 16-17
32	Describe any software that will be used during the consensus process and to analyse the results	Study overview<br>Page 14
33	Describe any plans for obtaining research ethics committee / institutional review board approval in relation to the consensus process (if relevant)	Declarations (Ethics approval and consent to participate)<br>Page 19
34	Describe how informed consent will be obtained (if relevant)	Study overview<br>Page 12-17
35	Describe any details about how the confidentiality of data collection will be preserved during the consensus process (if relevant)	Study overview<br>Page 16-17

For peer review only

Women's Interview schedule

Introduction to interview:

The aim of this interview is to understand your thoughts on what information you think is important to be discussed when you are pregnant and planning to have a vaginal birth. We will ask about your plans for your labour or experiences you have had. We would also like to hear about your thoughts on what information is important in order to make sure we can have the right discussions with women who are having a baby.

Topic area 1: What are you currently planning for your labour and birth?

Prompts: Location, how, pain relief, the room, the people present

Topic area 2: Tell me about your labour experience/birth? [IF POSTNATAL]

Prompts: Location, analgesia, people in room, birth plan

Topic area 3: Have you received any information about labour or vaginal birth antenatally?

Prompts: Resources used, antenatal classes, websites.

Topic area 4: Where would you look for information on vaginal birth?

Topic area 5: Have you heard of any potential risks of vaginal delivery?

Prompts: Risks during, risks after, anecdotes/stories heard

Topic area 6: Was there anything about vaginal birth that you wish you'd been told before you had your baby? [IF POSTNATAL]

Prompts: Misleading information prior, surprising aspects to delivery

Topic area 7: What information do you think is most important to know before having a vaginal birth?

Prompts: risks during, risks afterwards, long term effects, people who are in attendance

Topic area 8: Do you think women should be consented for a vaginal birth?

Prompts: should there be a form, what should be on it, who would the discussion be with

Topic area 9: Did you experience anything in your labour which you think is important to know about prior to having a baby?

Prompts: complications, waiting times, pain, long-term effects

Topic area 10: What are the key information points to know about a vaginal birth, in your opinion?

For peer review only - http://bmjopen.bmj.com/site/about/guidelines.xhtml

BMJ Open

Vaginal Birth Core Information Set: Study protocol for a Delphi study to achieve a consensus on a ‘core information set’ for vaginal birth

Journal:	*BMJ Open*
Manuscript ID	bmjopen-2022-070215.R1
Article Type:	Protocol
Date Submitted by the Author:	20-Mar-2023
Complete List of Authors:	Demetri, Andrew; University of Bristol,<br>Davies, Anna; University of Bristol<br>Bakhbakhi, Danya; University of Bristol<br>Ijaz , Sharea ; University of Bristol<br>Dawson, Sarah; University of Bristol<br>McGuinness, Sheelagh; University of Bristol<br>Beasor, Gemma; University of Bristol<br>Clayton, Gemma; University of Bristol<br>Johnson, Abigail; North Bristol NHS Trust<br>De Souza, Chloe; North Bristol NHS Trust<br>Dempsey, Aine; University Hospitals Bristol and Weston NHS Foundation Trust<br>Snook, Gabriella; North Bristol NHS Trust<br>Sharp, Andrew; University of Liverpool<br>Lissauer, David ; University of Liverpool<br>Mcgoldrick, Emma; Liverpool Women's Hospital<br>Burden, Christy; University of Bristol<br>Merriel, Abi; University of Liverpool
**Primary Subject Heading**:	Obstetrics and gynaecology
Secondary Subject Heading:	Evidence based practice, Ethics, Communication, Health policy, Qualitative research
Keywords:	OBSTETRICS, Maternal medicine < OBSTETRICS, Anaesthesia in obstetrics < ANAESTHETICS, MEDICAL ETHICS

SCHOLARONE™
Manuscripts

Title: Vaginal Birth Core Information Set: Study protocol for a Delphi study to achieve a consensus on a 'core information set' for vaginal birth

Authors

Dr Andrew Demetri, Academic Clinic Fellow, University of Bristol, andrew.demetri@bristol.ac.uk, https://orcid.org/0000-0002-2820-5919

Dr Anna Davies, Research Fellow, University of Bristol, anna.davies@bristol.ac.uk, https://orcid.org/0000-0003-0743-6547

Dr Danya Bakhbakhi, NIHR Doctoral Research Fellow, University of Bristol, danya.bakhbakhi@bristol.ac.uk

Dr Sharea Ijaz, Senior Research Associate in Evidence, University of Bristol, s.ijaz@bristol.ac.uk

Ms Sarah Dawson, Senior Research Associate in Information Retrieval, University of Bristol, sarah.dawson@bristol.ac.uk

Professor Sheelagh McGuinness, Reader in Law, University of Bristol, sheelagh.mcguiness@bristol.ac.uk

Ms Gemma Beasor, Patient representative, gemma.beasor@hotmail.co.uk

Dr Gemma Clayton, Senior Research Associate in Biostatistics, University of Bristol, gemma.clayton@bristol.ac.uk

Ms Abigail Johnson, Midwife, North Bristol NHS Trust, Abigail.Johnson@nbt.nhs.uk

Dr Chloe de Souza, Obstetrics and Gynaecology Trainee, North Bristol NHS Trust, chloe.desouza@nbt.nhs.uk

Dr Aine Dempsey, Obstetrics and Gynaecology Trainee, St Michael's Hospital, aine.dempsey@nhs.net

Dr Gabriella Snook, Obstetrics and Gynaecology Trainee, North Bristol NHS Trust, gabriella.snook@nbt.nhs.uk

Dr Andrew Sharp, Senior Clinical Lecturer in Obstetrics, University of Liverpool, asharp@liverpool.ac.uk

Professor David Lissauer, NIHR Professor of Global and Fetal Medicine, University of Liverpool, David.Lissauer@liverpool.ac.uk

Dr Emma McGoldrick, NIHR Research Scholar and Consultant Obstetrician, Liverpool Women's Hospital, EmmaLouise.McGoldrick@lwh.nhs.uk

For peer review only

Dr Christy Burden, Consultant Senior Lecturer, University of Bristol, christy.burden@bristol.ac.uk

Dr Abi Merriel, Senior Clinical Lecturer in Obstetrics, University of Liverpool, abi.merriel@liverpool.ac.uk, https://orcid.org/**0000-0003-0352-2106**

Corresponding author

Name: Dr Abi Merriel

Role: Senior Clinical Lecturer in Obstetrics

Postal address: University of Liverpool

Tel: 07740334922

Email: abi.merriel@liverpool.ac.uk

Word count: 3,999

For peer review only

Abstract

Introduction

Studies have shown that women are often underinformed about potential benefits and risks of vaginal birth. This is in contrast to other modes of birth, like caesarean birth, for which the risks/benefits are often conveyed prior to undergoing the procedure. A core information set (CIS) is an agreed set of information points that should be discussed with all patients prior to undergoing a procedure or intervention. This CIS could improve the quality of information given regarding mode of birth options, as women will be given information prioritised by patients and stakeholders regarding vaginal birth, empowering them to make informed decisions about their birth. We aim to describe the protocol for the development of this vaginal birth CIS.

Methods and analysis

We will develop the CIS by:

1. Compiling a 'long-list' of information points about vaginal birth by: undertaking a scoping review of studies and patient information leaflets; interviews with antenatal/postnatal women, an online survey of stakeholders.

2. Collating the 'long-list' of information points and developing the Delphi survey. Think-aloud interviews will refine the survey.

3. Conducting a two-round Delphi survey. 200 stakeholder participants will be recruited. Items rated critically important by ≥80% of participants in one stakeholder group, or with no consensus, will be carried through to a stakeholder consensus meeting to decide the final CIS.

Planned start date 1st June 2022. Planned end date 31st August 2023.

For peer review only

Ethics and dissemination

This project has been given a favourable ethics opinion by the University of Bristol Research Ethics Committee (Ref: 10530).

Approval from the ethics committee will be sought for any protocol amendments, and the principal investigator will be responsible for these changes.

Findings will be presented at relevant conferences and published in a high-impact journal.

We will disseminate the CIS, via Policy Bristol, to clinical policy and guideline developers.

Registration

This study is registered through the COMET initiative:

https://www.comet-initiative.org/Studies/Details/2069

Key words

Core information set, Delphi technique, Consensus, Informed consent, Vaginal birth, Stakeholders, Women, Women's health

For peer review only

For peer review only - http://bmjopen.bmj.com/site/about/guidelines.xhtml

Article Summary

Strengths and limitations of this study

- Patient involvement is central to the development of this body of work, meaning that the core information set will reflect the beliefs and thoughts of the women it will be helping.
- These methods, including the use of in-depth qualitative interviews, the Delphi survey and consensus process, means the development of this core information set will be robust and comprehensive, adding weight to the findings.
- The qualitative interviews, Delphi survey and consensus meetings will only be conducted in the English language due to funding and time limitations.
- The qualitative interviews to help with the formulation of the long list will only be with antenatal and postnatal women, and not the other stakeholder groups, however, their views will be incorporated using the online survey to ensure their views are not missing from the list.
- Due to time and financial constraints, in-depth qualitative interviews will only include women who have given birth in the last 5 years, so may miss some longer-term information points relating to vaginal birth.

For peer review only

Background

Spontaneous vaginal birth (SVB) accounts for approximately 50% of births in the United Kingdom.[1] Its benefits, risks and range of experiences are often not discussed in detail with women prior to them opting for a vaginal birth (VB). This is perhaps because VB is the presumed choice for women, unless they have an indication for, or request, a caesarean birth (CB). Recent work has looked at improving informed choice regarding birth method, as well as developing decision aids for women choosing between vaginal or CB.[2–4] To improve the quality of maternity care, further work must be done to ensure women are provided with the information they require to make a fully informed choice about their planned mode of birth.

VB is a physiological process and has benefits for mother and baby. Examples include shorter hospital stays, quicker recovery times and increased rates of breast feeding compared with CS.[5, 6] Risks of VB include shoulder dystocia (baby's shoulders getting stuck once its head has been born) which may lead to birth trauma or asphyxia, and 3rd or 4th degree tears which can result in longer term pelvic floor dysfunction.[7] These outcomes may have significant impact on women's long term physical and psychological functioning.[8]

However, even if a woman has made a positive informed choice for a VB, it may not be successful. In 2020-1, NHS statistics showed that 15% of women required an emergency instrumental delivery, and 19% an emergency CB, and most of these will have been aiming for a VB.[9] The risks of emergency CB are increased compared with elective CS, including having a higher incidence of wound infection and postpartum haemorrhage (PPH).[7, 10]

For peer review only - http://bmjopen.bmj.com/site/about/guidelines.xhtml

Likewise, instrumental deliveries carry higher risks than SVB, including increased risk of perineal trauma.[7, 10] Knowledge about VB, including risks and benefits, may influence women's choices on their mode of birth and birth experience. However, women are generally not explicitly offered information or this choice.

There is a lack of, and inconsistency in, information that women receive about VB.[5] This lack of information antenatally can contribute to poorer birth experiences by increasing anxieties with 'fear of the unknown'.[11, 12] The landmark Montgomery case,[13] good clinical practice and NHS improvement support the provision of information for patients to enable them to make truly informed decisions.[14],[15] By not providing this, healthcare professionals are failing in the professional, moral, and legal requirement, to support decision making about birth.

Women need access to consistent, accurate information, about what is important to them, when making birth choices. Whilst work has been done to improve information provision for women about CS,[16] very little has been done to understand what information women need about VB to make decisions about mode of birth. Core information sets (CIS) have been developed as a systematic means to provide information that is judged to be important by all relevant stakeholders about medical procedures. A CIS [17, 18] is a list of key information points regarding an experience that should be discussed with a person prior to experiencing an event or procedure. CIS are developed with the input of key groups including the patient, clinicians, and other relevant stakeholders, to identify the most important information.[19] A CIS differs from a core outcome set (COS), in that a COS is a set of outcomes that are measured or reported, as a minimum, in studies relating the subject. A CIS focuses on

For peer review only - http://bmjopen.bmj.com/site/about/guidelines.xhtml

information points that should discussed with patients. Despite these differences, the methodology for producing the respective sets is similar, allowing for adoption of the techniques.[19] Having a CIS to discuss with pregnant women will ensure that they are well informed about the decision they are making. Previous CIS have been used as catalysts for discussion about procedures, to improve content of information leaflets and improve decision aids.[17]

We aim to produce a CIS for VB that can be used to facilitate informed choice by improving discussions and enhancing tools used to help women with decision-making about birth choices. *This CIS will be useful for anyone planning, or considering having, a VB.* This will support preparation for VB, enabling better birth experiences and greater satisfaction, as well as enhancing women's post-natal wellbeing. Here we describe the protocol for development of the CIS.

Aim

To develop a CIS for vaginal birth to support informed decision making for childbirth.

Methods/Design

Design

We will conduct a systematic scoping review, qualitative interviews, and use a modified Delphi technique to identify the key information for the CIS. The Delphi process has been used in prior CIS studies,[17, 18] and involves multiple survey rounds of key stakeholder groups to condense the long list of information points, reaching a consensus on a CIS for VB.

For peer review only - http://bmjopen.bmj.com/site/about/guidelines.xhtml

Methods

This project has been given a favourable ethics opinion by the University of Bristol Research Ethics Committee (Ref: 10530).

The study is registered with COMET (https://comet-initiative.org/Studies/Details/2069) and reporting adheres to the Core Outcome Set-STAndards for Development (COS-STAD), and COS-STAndardised Protocol Items (COS-STAP) have been used to guide the development of the methods.[20, 21] A modified COS-STAP checklist for the VB CIS is included in the supplementary information (appendix S1). The COS-Standards for Reporting (COS-STAR) will be used to report the CIS.[22]

Whilst there is no standardised way to develop a CIS,[23] existing CIS have adapted methods from the development of COS.[17, 18] The Core Outcome Measures in Effectiveness (COMET) handbook and methods from existing CIS have been used to design this study.[17–19, 24]

SCOPE OF THE CIS

The CIS will be applicable to women who are considering SVB. SVB is an unassisted VB, without vacuum or forceps, with a natural onset not through medical intervention.[25, 26] We will exclude information points pertaining to the decision-making process of induction of labour, instrumental delivery, CB and vaginal birth after caesarean section (VBAC).

Patient and Public Involvement (PPI)

PPI is key to this study, with involvement of a wide range of stakeholder groups in the development and undertaking of this study. Women's perspectives, and those of their

For peer review only

partners, are pivotal to each stage of this study, including the qualitative interviews, Delphi survey and its development, and consensus meeting. The study steering committee contains patient representation, ensuring the development of the project is guided by those that the work will affect.

Study Overview

The development of this CIS will take place in four stages (figure 1: study overview diagram).

Stage 1: Identification of key information points.

A long list of information points pertaining to VB will be generated through three methods:

a) Scoping review of the literature: Information points about VB from published studies will be extracted as well as from patient information leaflets provided to women in the NHS and other trusted organisations.
b) Interviews with women: qualitative interviews will be conducted with pregnant and postnatal women to determine what information is valued by women.
c) Stakeholder survey: An online survey of stakeholders (women, partners, healthcare professionals, interested groups and medico-legal experts) to identify important information for women.

a) Scoping review

Search: Information about VB will be identified from systematic reviews of pregnancy and childbirth literature and supplemented by a review of patient information leaflets. A pragmatic search strategy will be developed by an information specialist to identify

For peer review only

For peer review only - http://bmjopen.bmj.com/site/about/guidelines.xhtml

outcomes reported in studies, and key issues women identify, about VB. Studies included in the review will be systematic reviews, both qualitative and quantitative. Search terms will include vaginal birth, vaginal delivery, birth, childbirth, natural childbirth, labour, labor, parturition. Searches will be applied to the following electronic databases: CINAHL, Embase, MEDLINE, Web of Science, DoPHER, Epistemonikos, Health Evidence and the Cochrane Library. Searches will be limited to systematic reviews published in the English language. The date is limited to 2020 to 2022 for the papers, and to 2017 to 2022 for the Cochrane reviews, due to the large volume of papers that would meet the criteria, thus meaning data saturation from the literature is likely to be achieved within this sample.

We will search for patient information leaflets on VB, to extract information points from these. We will search the online leaflet collections of the Royal College of Obstetricians and Gynaecologists, Tommy's Pregnancy Information and NHS trusts with an online database of leaflets. Leaflets relating to assisted vaginal birth (AVB) or VBAC will be excluded from this review, as items relating to these are likely to differ from those for SVB and we hope to develop separate CIS for these in future.

Paper selection: Screening of titles, abstracts and full texts will be carried out by two members of the research team using systematic reviewing software (Covidence).[27] Any disagreements will be resolved by a third reviewer. We will include all qualitative, quantitative, and mixed-method systematic reviews that include information points or outcomes for VB. No studies will be excluded based on judgement of methodological quality or risk of bias assessment as the purpose of the scoping review is to identify information points and outcomes relating to VB, not to examine the quality of data.

For peer review only

Data extraction: Data will be extracted using a pre-designed form, piloted by two members of the research team on a sample of included studies. Data will include basic publication details such as author, publication date, study setting and methodology. A pre-designed, and piloted, form will be used for data extraction from leaflets. Extracted data will include format of leaflet, source, and year of publication. Outcomes and information points will be extracted verbatim. It will not be necessary to conduct a risk of bias analysis.

b) Qualitative interviews

Method: Semi-structured interviews

Sample: We will recruit up to 20 women who are >12 weeks pregnant, and/or have had a baby, including those who have experienced an intrauterine death (diagnosed pre-labour or during labour), in the last 24 months. Sample size for these interviews will be determined by data saturation being reached, whereby interviews will end when no new themes are generated. Women will be recruited online, via social media and by adverts in community centres. Initial screening questions regarding mode of birth will be devised to allow for purposive sampling of women with a range of birth experiences and preferences including VB, CB and AVB. This will ensure that we gather information points from women from diverse stand points in terms of their decision-making journey to VB. Those who are unable to speak English or consent will be excluded from recruitment due to lack of funds for translation.

Interviews: Informed consent will be gained using an online form, hosted on REDCap.[28] A topic guide will be developed with the study steering group (appendix S2). Interviews will be conducted by a qualitative researcher, recorded on an encrypted audio recorder, and

For peer review only - http://bmjopen.bmj.com/site/about/guidelines.xhtml

uploaded on to the secure University of Bristol server. At the start of the interview, demographic questions will be asked. Participants will receive a £10 voucher to compensate for their time and inconvenience.

Analysis: Audio recordings of the interviews will be transcribed, and analysed using NVivo software, using thematic analysis.[29] Two members of the research team will read the transcripts initially to familiarise themselves with the process. Interviews will be independently coded by the two members of the research team, to identify important information points about VB from the words of participants. Transcripts will be re-read, and each researcher's codes compared for each interview. Codes will then be grouped to create key themes, and key information points will be identified from each of these.[30] Analysis whilst the interviews are underway will ensure that when data saturation is reached, no additional interviews will be conducted unnecessarily.[31–33]

c) Stakeholder survey:

Method: An online qualitative survey will be used to identify further information items by gathering key stakeholder views on what information is important for women to receive about VB.

Sample: Stakeholders include antenatal and postnatal women, their partners, healthcare professionals who work alongside women in labour and postnatally, representatives from groups with an interest in women's birthing rights and medico-legal experts with an interest in childbirth. We will aim to engage approximately 200 survey participants, recruited online through social media and in community spaces. This sample size has been decided upon to

For peer review only

ensure that a wide range of views and experiences are captured. We will aim for half of the responses to be from parents whose experiences and views are central to this work.

Data collection: The survey will be carried out online using REDCap.[28] The survey will include information on the participant's role and demographics, including if they have previously experienced a VB.

To identify what information they believe is vital to provide to women about VB,

a free text box will be used. All participants in the survey will be invited to participate in the Delphi process. Participants will be entered into a prize draw to win a voucher worth £10 (5 vouchers available).

Analysis: Survey data will be thematically analysed by two researchers and key information points extracted. These information points will be added to those from the scoping review and interviews.

Stage 2: Developing the long list of information points and Think-aloud Interviews.

Developing the long list of information points

Following the identification of key points, we will produce an exhaustive list of information items relating to VB, known as a 'long list', which will feed into the initial stage of the Delphi process.[34, 35] Two members of the team will use all information items from the scoping review, interviews, and stakeholder survey to generate the comprehensive list. Information items will be organised into categories for coherence. Examples of categories for information points which may be included are for points relating to maternal physical health, maternal mental health, or baby health. Categories may be removed or created dependent on the information points that are discovered.

For peer review only - http://bmjopen.bmj.com/site/about/guidelines.xhtml

A meeting will be held to finalise the long list for the Delphi process and establish how the items should be presented and worded in the Delphi survey. This meeting will involve members of the research team. The research team is made up of doctors, midwives, medico-legal experts, a patient representative, and researchers. During this meeting, categories will be discussed for each item. Additionally, definitions will be decided to ensure they are understandable for all participants. Where items are similar, group discussions and decisions will take place to ensure there is no duplication.

For peer review only

Piloting the Delphi survey

Up to 10 think-aloud interviews will be undertaken to test the usability of the Delphi survey and refine the long list of items.[36] The think-aloud method is a well-recognised validation technique for developing an instrument or tool, and has been used by COS developers to improve Delphi questionnaires.[19, 37] This method is a form of cognitive interviewing that asks participants to verbalise their thoughts as they engage with a task, in this case the survey.[38] We will observe how participants interpret the information items and definitions, and how they interact with the scoring system, identifying any issues. Participants' thoughts are used to improve and perfect the survey to ensure it is understandable and easy to complete. We will record the length of time the survey takes, ensuring it is not too long, therefore minimising answer fatigue and maximising responses.[19]

After completing a consent form, participants will be asked to trial the Delphi survey using the online platform. Interviews will last thirty minutes to one hour and take place online via teleconferencing software. The interviews will be audio recorded on a University of Bristol (UoB) encrypted device, and the interviewer will take notes. 2-3 initial interviews will take place, and comments regarding the survey will be noted, with the audio recordings used if

For peer review only - http://bmjopen.bmj.com/site/about/guidelines.xhtml

needed to remind about key points. The comments will then be used to improve the survey, before carrying out the next 2-3 surveys and repeating the process. The approach is repeated until no further changes are indicated, which we estimate will be after approximately 10 interviews.

Stage 3: The Delphi Survey

Delphi overview

We will aim to conduct two iterative Delphi rounds, followed by a consensus meeting. The survey will be conducted online using REDCap hosted by the University of Bristol.[28]

Participants and sample size

Participants in the Delphi process will include:

1) Women and their partners:

   Any woman who is over the age of 18, who is currently pregnant, or has had a baby in the last 5 years, and partners of these women.

2) Healthcare professionals

   Those who work with, or provide care for, women, during their pregnancy, during childbirth, and/or postnatally (e.g. obstetricians and gynaecologists, including trainees, midwives, midwifery support workers, physiotherapists, anaesthetists, general practitioners).

3) Medicolegal experts

   Legal professionals with a specialist interest in reproductive health.

4) Representatives from interested groups/stakeholders

For peer review only

Representatives from organisations/groups with an interest in women's birthing rights (e.g. Birthrights, Maternity Action).

Sample size: We will obtain a sample with a broad range of experience, an approach supported by the COMET guidelines and previous studies.[17, 24] There is no specified size of stakeholder sample for CIS or COS studies.[18, 23, 24] Some have estimated that the drop-out rate for a Delphi process is around 20%.[35] To account for this potential attrition, we will aim to engage at least 200 participants in the Delphi process. We will aim for at least half of these participants to be antenatal and postnatal women, as their views are vital for this CIS. When gathering responses, if we feel some stakeholder groups are underrepresented, more targeted recruitment for the survey will take place to increase representation.

Delphi rounds

A two-round modified Delphi process will be conducted online, with each round lasting for 2-8 weeks, dependent on engagement.[17, 28] Participants will be asked to complete a consent form before completing the survey, as well as demographic information. Participants will score each survey item for importance for inclusion from 1-9 (1-3- limited importance; 4-6- important but not critical; 7-9- critical).[24, 39] A-priori consensus criteria will be applied.[34] Information items to be carried through to the subsequent round will require ≥80% of participants from either patient/representative group (women, partners, representatives of interested groups) or the professional group (healthcare workers, medico-legal experts) to score it as critically important (7-9) and <15% from any group to classify it as limited importance. Items will not be carried forward if ≥80% of one group

For peer review only - http://bmjopen.bmj.com/site/about/guidelines.xhtml

believe it is of limited importance, and <15% believe it is critically important. Where items are rated as critical by ≥80% of participants in one stakeholder group, or there is no consensus, they will be carried forward to the next round.

In round two participants who took part in the first survey will be asked to recomplete the survey. They will be informed of their own scores and the median scores of patient/representative and professional groups using both numeric data and graphical illustrations of theirs and others' responses. Items carried through to the consensus meetings will be selected based on the above criteria. If many of the information points meet the criteria to be carried forward to either the 2nd Delphi round or the consensus meeting, the steering group will meet to review the criteria. The steering committee consists of doctors, midwives, researchers, reproductive health lawyer and a patient representative.

In this situation, an adapted form of the OMECRACT framework may be used to organise items.[40, 41] Based on this methodology, if many points meet the inclusion criteria, items will be grouped into categories to determine importance and need to be included. Concentric circles indicate the hierarchy of information points (figure 2: OMECRACT framework used to organise information items). The central ring will be 'absolutely vital information to be included in the CIS'. A further two groups for information points will be used, with a middle ring being 'useful information to have available but may not be vital for inclusion' and an outer ring for items that 'may or may not need to be discussed dependent on the personal circumstances or preferences.' If many items are retained following the application of this criteria, then holding more than one consensus meeting will be considered to ensure adequate time to discuss all points.

For peer review only

We will determine the level of attrition bias by comparing scores of stakeholders who completed both rounds with scores of those who completed only the first round.[19] For transparency, we will record and report the number who have only completed the first round.

Stage 4: The consensus meeting to decide the final core information set.

Consensus meeting

Following the second Delphi round, an online consensus meeting, using teleconferencing software, will take place with all participants from the second round of the Delphi invited. If a large number of the invited stakeholders accept the invitation, we will aim to have the minimum representation from each stakeholder group.[19] Screening questions will allow for purposive sampling to ensure diverse representation of views within these groups. We will aim to recruit 8-10 participants from each stakeholder group, as this number has been sufficient in previous COS.[19, 42] A modified nominal technique will be used as a template to drive discussions and reach a group consensus allowing for *all* participants' views to be heard in the discussion.[43]

During this meeting, items carried through from the second Delphi round will be discussed and descriptive statistics created for each domain and displayed to participants, to stimulate discussion, with median scores for each item presented as graphs.[18][34]

The participants will anonymously rate the retained information domains from the second round using online voting software.[18] Items will be categorised as 'Consensus in- information point to be included in final CIS', 'Consensus out- item not included in the final CIS' or 'No consensus- items for which opinions on inclusion are divided'. Consensus will be defined as ≥80% of participants agreeing on the vote for an item. Where consensus is not reached,

For peer review only

For peer review only - http://bmjopen.bmj.com/site/about/guidelines.xhtml

there will be further discussion and additional voting. Scores of those participating in the consensus meeting will be compared to those who only participated in the first two Delphi rounds, to assess if the consensus meeting views are representative of those who only completed the survey.[19] When consensus is reached for all items, we will have the final CIS.

There is no definitive amount of information points that should be included in a CIS. Of the published CIS, the number of information points included in the final CIS have ranged from 8 to 13.[17, 18] We will therefore aim to have a maximum of 15 information points in our CIS. Whilst it is important the list is concise, we recognise that the unique nature of VB means we may end up with more than 15 points, so there will be flexibility in the acceptable size of the list.

Discussion

Vaginal birth is physiological and carries benefits but is not without risk. Informed consent requires that women are well informed when making birth choices. We will develop a CIS using a systematic, transparent, process to help to standardise the information given to women planning to have a VB. It will be informed by what women and other stakeholders believe is the most important information to be discussed, which, if taken up, will ensure all have equitable access to this information.

This will be the first published CIS regarding VB. The study's biggest strength is PPI which is central to the work, both in study development and methodology, meaning the CIS will represent the voices of the women it is aiming to empower. Unfortunately, due to time and financial constraints, interviews to develop the long list will only include women who speak English and those who have given birth in the past 5 years, potentially missing some

For peer review only

480 information items. Other stakeholder groups will also not be interviewed. As this study aims to represent women's voices, interviewing women seems most appropriate. The scoping review and survey should mitigate this limitation, by identifying long-term outcomes, and representing other groups.

This CIS can be used as the catalyst for discussions about VB, to promote truly informed birth choices. With this CIS we will have worked out what information is important. Future work will be needed to elaborate on the information points, adding statistical data to support understanding of risks and benefits, from national databases, systematic literature reviews and expert input. The CIS can also be used to inform patient information leaflets and decision-making tools.

Ethics and Dissemination

The dissemination strategy for the CIS will be developed with the steering committee and Policy Bristol to ensure it is maximally utilised to improve discussions women have with healthcare professionals regarding their birth choices. The findings of the CIS will be published in a peer-reviewed specialty journal. An overview of the CIS will be shared with the COMET initiative, with whom the study is registered. The results will be presented at both national and international conferences of relevant bodies.

For the purpose of open access, the author has applied a Creative Commons 124 Attribution (CC BY) licence to any Author Accepted Manuscript version arising.

Ethical approval has been granted by the University of Bristol Research Ethics Committee (Ref: 10530).

Trial status

This is protocol version 1.1, written on 14th March 2022.

Competing interests

The authors of this paper have no competing interest.

Funding

This study was funded by the Research England Policy Support Fund (PSF). The sponsors have played no role in designing the study, data collection, analysis, interpretation of data, writing of the report and decisions for publication.

Author contributions

AD and AM conceived the study and obtained the funding. AD drafted the protocol and manuscript. AM, ADa, DB, CB, AS, DL and EM helped with drafting the manuscript and formulating the methods for the study. SD, SI, SM, CdS, GC, ADemp, and GS assisted with conceiving stage 1. GB, GC, and AJ helped with study conception and feasibility. All authors read and approved the final manuscript.

Authors legend:

AD- Andrew Demetri

AM- Abi Merriel

ADa- Anna Davis

DB- Danya Bakhbakhi

CB- Christy Burden

AS- Andrew Sharp

For peer review only

DL- David Lissauer

EM- Emma McGoldrick

SD- Sarah Dawson

SI- Sharea Ijaz

SM- Sheelagh McGuinness

CdS- Chloe de Souza

ADemp- Aine Dempsey

GS- Gabriella Snook

GB- Gemma Beasor

GC- Gemma Clayton

AJ- Abigail Johnson

<u>Acknowledgements</u>

We would like to thank the Research England Policy Support Fund (PSF) for funding this project.

<u>Data statement</u>

As this is a qualitative study, we do not intend on making the dataset publicly accessible. The data set will be put into the data.bris repository, with requests for access coming through this. The level of access will be controlled.

All members of the core study team will have access to this data. Individuals in the wider steering group will receive parts of the data, as required, for analysis and discussion, and can access the full dataset by visiting UoB.

For peer review only

It is not envisaged that this data will be used for analysis outside the remit of this study, however, we will ask permission to use the data for subsequent analysis in the consent form.

List of abbreviations

AVB: Assisted vaginal birth

CB: Caesarean birth

CIS: Core information set

COS-STAD: Core Outcome Set-STAndards for Development (COS-STAD)

COS-STAP: COS-STAndardised Protocol Items (COS-STAP)

COS-STAR: COS-Standards for Reporting (COS-STAR)

PPH: Postpartum haemorrhage

PPI: Patient and public involvement

SVB: Spontaneous vaginal birth

VB: Vaginal birth

VBAC: Vaginal birth after caesarean section

Figures

Figure 1: Study overview diagram. Diagram showing the individual stages of the study and how they feed into the next. Also demonstrates where PPI and stakeholder input will be key in the study.

Figure 2: Omeract onion framework. This diagram demonstrates the framework which framework may be used to organise information items discussed at the consensus meeting.

For peer review only

Supplementary materials

Appendix S1- COS-STAP Checklist

Appendix S2- Antenatal and postnatal interview topic guide

References

[1] Maternity Services Monthly Statistics, February 2021, https://digital.nhs.uk/data-and-information/publications/statistical/maternity-services-monthly-statistics/february-2021 (2021, accessed 21 February 2022).
[2] Kingdon C, Neilson J, Singleton V, et al. Choice and birth method: mixed-method study of caesarean delivery for maternal request. *BJOG* 2009; 116: 886–895.
[3] Frost J, Shaw A, Montgomery A, et al. Women's views on the use of decision aids for decision making about the method of delivery following a previous caesarean section: qualitative interview study. *BJOG* 2009; 116: 896–905.
[4] Farnworth A, Robson SC, Thomson RG, et al. Decision support for women choosing mode of delivery after a previous caesarean section: A developmental study. *Patient Educ Couns* 2008; 71: 116–124.
[5] Shub A, Williamson K, Saunders L, et al. Do primigravidae and their carers have a realistic expectation of uncomplicated labour and delivery? *Australian and New Zealand Journal of Obstetrics and Gynaecology* 2012; 52: 73–77.
[6] Sandall J, Tribe RM, Avery L, et al. Short-term and long-term effects of caesarean section on the health of women and children. *The Lancet* 2018; 392: 1349–1357.
[7] Gregory K, Jackson S, Korst L, et al. Cesarean versus Vaginal Delivery: Whose Risks? Whose Benefits? *Am J Perinatol* 2012; 29: 07–18.
[8] Ford E, Ayers S. *Post-Traumatic Stress Disorder following Childbirth*. 2012.
[9] NHS Digital. Maternity Services Monthly Statistics, February 2022, experimental statistics, https://digital.nhs.uk/data-and-information/publications/statistical/maternity-services-monthly-statistics/february-2022-experimental-statistics (2022, accessed 21 June 2022).
[10] Darnal N, Dangal G. Maternal and Fetal Outcome in Emergency versus Elective Caesarean Section. *J Nepal Health Res Counc* 2020; 18: 186–189.
[11] Gammie N, Key S. Time's up! Women's experience of induction of labour. *Pract Midwife* 2014; 17: 15–8.
[12] Lavender T, Walkinshaw SA, Walton I. A prospective study of women's views of factors contributing to a positive birth experience. *Midwifery* 1999; 15: 40–46.
[13] Chan SW, Tulloch E, Cooper ES, et al. Montgomery and informed consent: where are we now? *BMJ* 2017; j2224.
[14] Whitney SN. A New Model of Medical Decisions: Exploring the Limits of Shared Decision Making. *Medical Decision Making* 2003; 23: 275–280.
[15] Britain G. *Maternity matters: choice, access and continuity of care in a safe service. Department of Health.* April 2007.
[16] Briscoe KE, Haas DM. Developing a Core Outcome Set for Cesarean Delivery Maternal Infectious Morbidity Outcomes. *Am J Perinatol* 2020; 37: 436–452.
[17] Blazeby JM, Macefield R, Blencowe NS, et al. Core information set for oesophageal cancer surgery. *British Journal of Surgery* 2015; 102: 936–943.

For peer review only

For peer review only - http://bmjopen.bmj.com/site/about/guidelines.xhtml

[18] McNair AGK, Whistance RN, Main B, et al. Development of a core information set for colorectal cancer surgery: a consensus study. *BMJ Open* 2019; 9: e028623.

[19] Bakhbakhi D, Fraser A, Siasakos D, et al. Protocol for the development of a core outcome set for stillbirth care research (iCHOOSE Study). *BMJ Open* 2022; 12: e056629.

[20] Kirkham JJ, Davis K, Altman DG, et al. Core Outcome Set-STAndards for Development: The COS-STAD recommendations. *PLoS Med* 2017; 14: e1002447.

[21] Kirkham JJ, Gorst S, Altman DG, et al. Core Outcome Set-STAndardised Protocol Items: the COS-STAP Statement. *Trials* 2019; 20: 116.

[22] Kirkham JJ, Gorst S, Altman DG, et al. Core Outcome Set–STAndards for Reporting: The COS-STAR Statement. *PLoS Med* 2016; 13: e1002148.

[23] Williamson PR, Altman DG, Blazeby JM, et al. Developing core outcome sets for clinical trials: issues to consider. *Trials* 2012; 13: 132.

[24] Williamson PR, Altman DG, Bagley H, et al. The COMET Handbook: version 1.0. *Trials* 2017; 18: 280.

[25] Prosser SJ, Barnett AG, Miller YD. Factors promoting or inhibiting normal birth. *BMC Pregnancy Childbirth* 2018; 18: 241.

[26] Clark RRS, Lake ET. Spontaneous vaginal birth varies significantly across US hospitals. *Birth* 2021; 48: 44–51.

[27] Covidence systematic review software. Melbourne, Australia: Veritas Healthth Innovation.

[28] Harris PA, Taylor R, Thielke R, et al. Research electronic data capture (REDCap)—A metadata-driven methodology and workflow process for providing translational research informatics support. *J Biomed Inform* 2009; 42: 377–381.

[29] Braun V, Clarke V. Using thematic analysis in psychology. *Qual Res Psychol* 2006; 3: 77–101.

[30] Clarke V, Braun V. Teaching thematic analysis: Overcoming challenges and developing strategies for effective learning. *Psychologist* 2013; 26: 120–123.

[31] Patton MQ. Two Decades of Developments in Qualitative Inquiry. *Qualitative Social Work* 2002; 1: 261–283.

[32] Connelly LM, Peltzer JN. Underdeveloped Themes in Qualitative Research. *Clinical Nurse Specialist* 2016; 30: 52–57.

[33] Sandelowski M. Sample size in qualitative research. *Res Nurs Health* 1995; 18: 179–183.

[34] dos Santos F, Drymiotou S, Antequera Martin A, et al. Development of a core outcome set for trials on induction of labour: an international multistakeholder Delphi study. *BJOG* 2018; 125: 1673–1680.

[35] Hall DA, Smith H, Heffernan E, et al. Recruiting and retaining participants in e-Delphi surveys for core outcome set development: Evaluating the COMiT'ID study. *PLoS One* 2018; 13: e0201378.

[36] Drennan J. Cognitive interviewing: verbal data in the design and pretesting of questionnaires. *J Adv Nurs* 2003; 42: 57–63.

[37] Messick S. *Validity*. 3rd ed. New York: American Council on Education/Macmillan Publishing, 1989.

[38] Ericsson KA, Simon HA. How to Study Thinking in Everyday Life: Contrasting Think-Aloud Protocols With Descriptions and Explanations of Thinking. *Mind Cult Act* 1998; 5: 178–186.

[39] Grade Working Group. GRADE, http://www.gradeworkinggroup.org (accessed 9 May 2022).

For peer review only

For peer review only - http://bmjopen.bmj.com/site/about/guidelines.xhtml

[40] Mackie SL, Twohig H, Neill LM, et al. The OMERACT Core Domain Set for Outcome Measures for Clinical Trials in Polymyalgia Rheumatica. *J Rheumatol* 2017; 44: 1515–1521.
[41] Humphrey-Murto S, Crew R, Shea B, et al. Consensus Building in OMERACT: Recommendations for Use of the Delphi for Core Outcome Set Development. *J Rheumatol* 2019; 46: 1041–1046.
[42] Perry H, Duffy JMN, Umadia O, et al. Outcome reporting across randomized trials and observational studies evaluating treatments for twin-twin transfusion syndrome: systematic review. *Ultrasound in Obstetrics & Gynecology* 2018; 52: 577–585.
[43] Harvey N, Holmes CA. Nominal group technique: An effective method for obtaining group consensus. *Int J Nurs Pract* 2012; 18: 188–194.

For peer review only

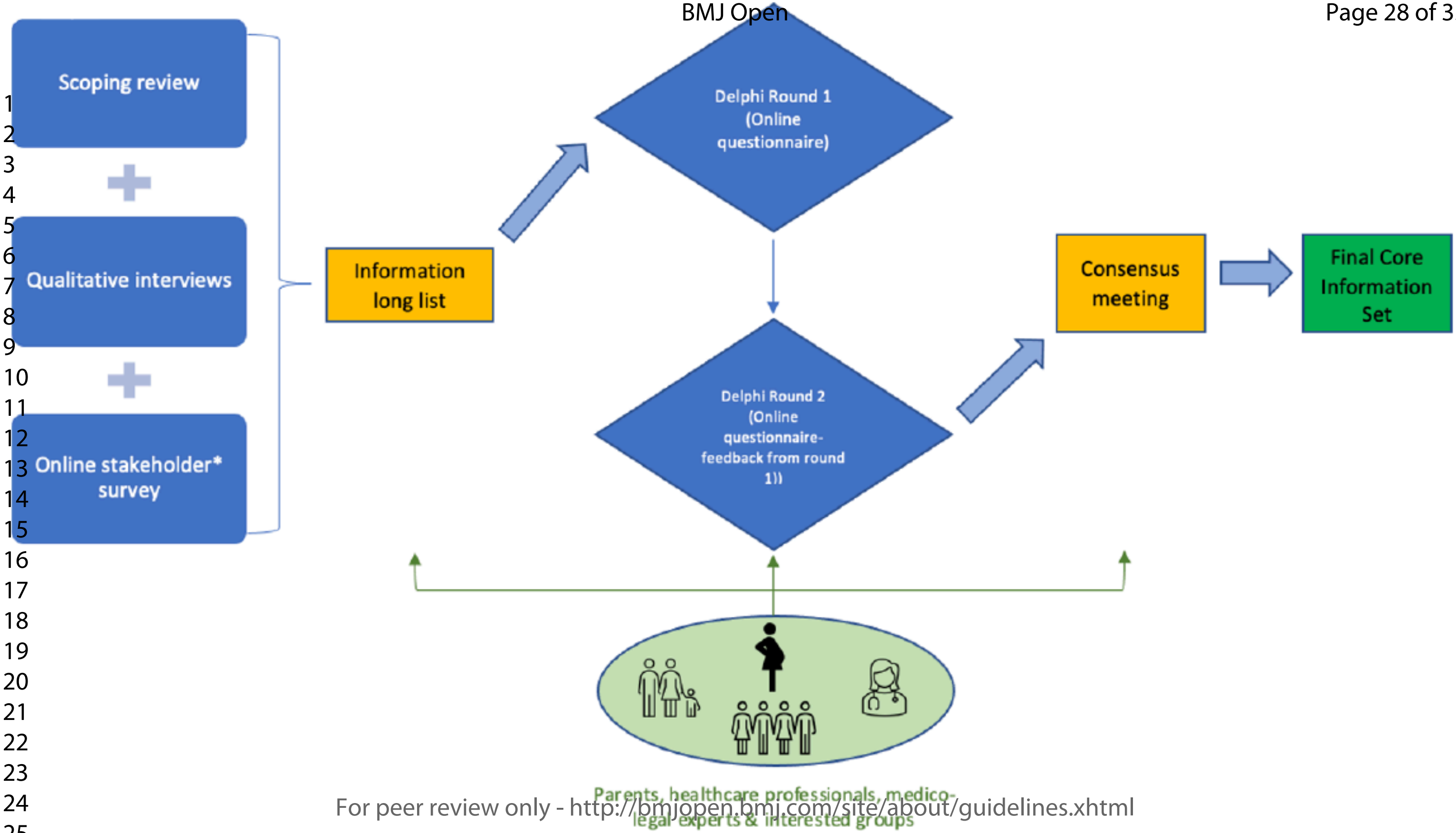

*Stakeholders include antenatal and postnatal women, their partners, healthcare professionals, interested groups and medico-legal experts

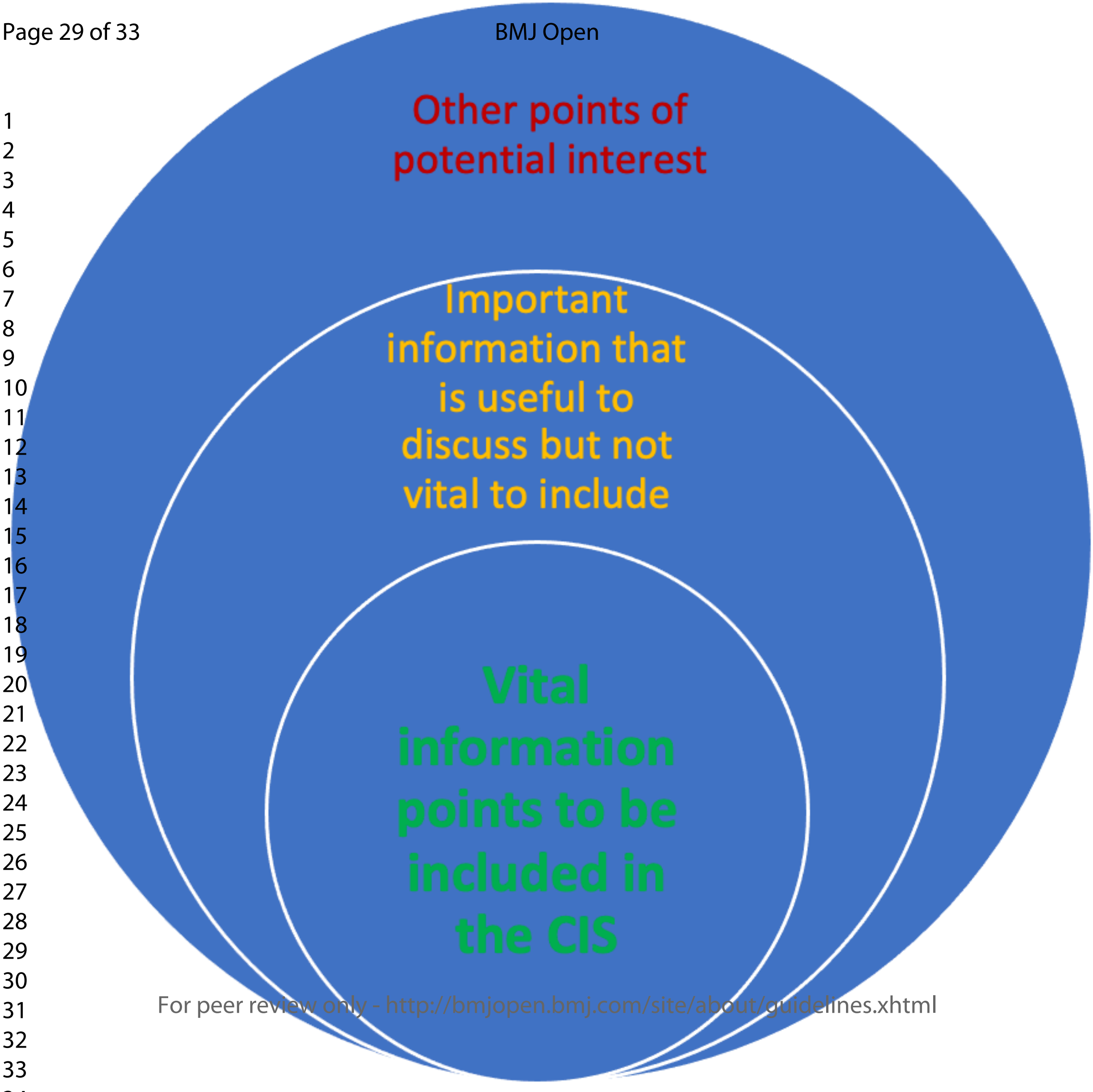
Other points of potential interest
Important information that is useful to discuss but not vital to include
Vital information points to be included in the CIS

Core Outcome Set-STAndardised Protocol Items (COS-STAP) Checklist for the 'Vaginal Birth Core Information Set' Study

Item Number	Name	Location
1	Identify in the title that the paper describes the protocol for the planned development of a CIS	Title/Abstract<br>Page 1-2
2	Provide a structured abstract	Abstract<br>Page 2-3
3	Describe the background and explain the rationale for developing the CIS	Background<br>Page 4
4	Describe the specific objectives with reference to developing a CIS	Background and Aim<br>Page 4-6
5	Describe the health condition(s) and population(s) that will be covered by the CIS	Methods/Design (Scope of CIS)<br>Page 7
6	Describe the intervention(s) that will be covered by the CIS	Methods/Design<br>Page 7
7	Describe the setting(s) that will be covered by the CIS	Methods/Design<br>Page 7
8	Indicate the CIS study registration details and registry name. If not yet registered indicate the intended registry	Methods/Design<br>Page 7
9	Describe any study oversight committees	Oversight and monitoring (Trial steering committee)<br>Page 18
10	Describe sources of funding, role of funders	Declarations (Funding)<br>Page 20
11	Describe any potential conflicts of interest within the study team and how these will be managed	Declarations (Competing interests)<br>Page 20
12	Describe the stakeholder groups to be involved in the CIS development process and the rationale for their involvement	Oversight and monitoring (Trial steering committee)<br>Page 18
13	Describe the eligibility criteria for individuals from each stakeholder group	Study overview<br>Page 10-14
14	Describe how individuals of each stakeholder groups will be identified	Study overview<br>Page 10-14

For peer review only

15	Describe how individuals of each stakeholder group will be chosen from within the stakeholder group	Study overview<br>Page 10-14
16	Describe how many planned individuals within each stakeholder group will be invited to participate in the consensus process	Study overview<br>Page 10-14
17	Describe how individuals will be invited to take part in the consensus process	Study overview (Stage 4)<br>Page 16
18	Describe the information sources that will be used to identify the list of outcomes. Outline the methods or reference other protocols/papers.	Study overview (Stage 1)<br>Page 8-11
19	Describe how outcomes may be dropped/combined, with reasons	Study overview Study overview (Stage 2)<br>Page 12
20	Describe the methods to identify outcome descriptor terms	Study overview (Stage 2)<br>Page 12
21	Describe the plans for how the consensus process will be undertaken	Study overview (Stage 4)<br>Page 16
22	Describe what information will be presented to participants at the start of the consensus process	Study overview (Stage 4)<br>Page 16
23	Describe what each participant will be asked to do at each stage of the consensus process	Study overview (Stage 4)<br>Page 16
24	Describe how the participants will receive any feedback during the consensus process	Study overview (Stage 4)<br>Page 16-17
25	Describe how non-response (or partial response) will be handled during the consensus process	Study overview (Stage 4)<br>Page 16
26	Describe how the study material will be made patient friendly and understandable (if relevant)	Study overview (Think-aloud interviews)<br>Page 12
27	Describe the consensus definition	Study overview (Delphi rounds)<br>Page 15
28	Describe the procedure for determining how outcomes will be added/combined/dropped from consideration during the consensus process	Study overview (Delphi rounds)<br>Page 15
29	Describe how outcomes will be scored and summarised	Study overview (Delphi rounds)<br>Page 15

30	Describe how the response rate will be maximised	Study overview (Piloting the Delphi survey)<br>Page 13
31	Describe how attrition bias will be assessed	Study overview<br>Page 16-17
32	Describe any software that will be used during the consensus process and to analyse the results	Study overview<br>Page 14
33	Describe any plans for obtaining research ethics committee / institutional review board approval in relation to the consensus process (if relevant)	Declarations (Ethics approval and consent to participate)<br>Page 19
34	Describe how informed consent will be obtained (if relevant)	Study overview<br>Page 12-17
35	Describe any details about how the confidentiality of data collection will be preserved during the consensus process (if relevant)	Study overview<br>Page 16-17

For peer review only

University of BRISTOL

Women's Interview schedule

Introduction to interview:

The aim of this interview is to understand your thoughts on what information you think is important to be discussed when you are pregnant and planning to have a vaginal birth. We will ask about your plans for your labour or experiences you have had. We would also like to hear about your thoughts on what information is important in order to make sure we can have the right discussions with women who are having a baby.

Topic area 1: What are you currently planning for your labour and birth?

Prompts: Location, how, pain relief, the room, the people present

Topic area 2: Tell me about your labour experience/birth? [IF POSTNATAL]

Prompts: Location, analgesia, people in room, birth plan

Topic area 3: Have you received any information about labour or vaginal birth antenatally?

Prompts: Resources used, antenatal classes, websites.

Topic area 4: Where would you look for information on vaginal birth?

Topic area 5: Have you heard of any potential risks of vaginal delivery?

Prompts: Risks during, risks after, anecdotes/stories heard

Topic area 6: Was there anything about vaginal birth that you wish you'd been told before you had your baby? [IF POSTNATAL]

Prompts: Misleading information prior, surprising aspects to delivery

Topic area 7: What information do you think is most important to know before having a vaginal birth?

Prompts: risks during, risks afterwards, long term effects, people who are in attendance

Topic area 8: Do you think women should be consented for a vaginal birth?

Prompts: should there be a form, what should be on it, who would the discussion be with

Topic area 9: Did you experience anything in your labour which you think is important to know about prior to having a baby?

Prompts: complications, waiting times, pain, long-term effects

Topic area 10: What are the key information points to know about a vaginal birth, in your opinion?

Version 1.1 27/02/22

For peer review only - http://bmjopen.bmj.com/site/about/guidelines.xhtml
